# Macroscopic to ultrastructural analyses identify the loss of myofibrils as the primary mediator of ageing‐ and disuse‐induced muscle fibre atrophy

**DOI:** 10.1113/JP291728

**Published:** 2026-09-25

**Authors:** Ramy K. A. Sayed, Anthony N. Lange, Hector G. Paez, Jamie E. Hibbert, Marius Meinhold, Corey G. K. Flynn, Jared Hartung, Matilde B. Z. Santibanez, Isabell Dobrzycki, David J. Wrucke, Carlos S. Zepeda, Jessica J. James, Christopher W. Sundberg, Troy A. Hornberger

**Affiliations:** ^1^ Department of Comparative Biosciences University of Wisconsin‐Madison Madison Wisconsin USA; ^2^ School of Veterinary Medicine University of Wisconsin‐Madison Madison Wisconsin USA; ^3^ Department of Anatomy and Embryology Faculty of Veterinary Medicine Sohag University Sohag Egypt; ^4^ Exercise and Rehabilitation Sciences Program, Department of Physical Therapy Marquette University Milwaukee Wisconsin USA; ^5^ Division of Geriatrics and Gerontology Department of Medicine University of Wisconsin–Madison Madison Wisconsin USA; ^6^ Department of Kinesiology University of Wisconsin–Madison Madison Wisconsin USA; ^7^ Athletic and Human Performance Research Center Marquette University Milwaukee Wisconsin USA

**Keywords:** atrophy, FIM‐ID, immobilization, muscle fibre, myofibrils, sarcopenia

## Abstract

**Abstract:**

Ageing and disuse are two of the most clinically relevant conditions associated with the loss of skeletal muscle mass, yet the ultrastructural adaptations that drive these losses remain poorly defined. Indeed, even the most basic questions, such as whether radial atrophy of muscle fibres is driven primarily by reductions in myofibril size, and/or the loss of myofibrils, remain unanswered. To address this gap, skeletal muscle structure was assessed at the macroscopic, microscopic and ultrastructural levels in young and older humans and mice. In humans, magnetic resonance imaging was used to measure quadriceps muscle volume and cross‐sectional area (CSA), whereas vastus lateralis biopsies underwent standard immunohistochemistry for microscopic evaluations, coupled with a next‐generation fluorescence imaging pipeline for ultrastructural analyses. Parallel experiments were conducted in mice, including a unilateral immobilization model of disuse‐induced atrophy. Ageing in humans was associated with lower muscle volume and CSA, along with radial atrophy of SERCA1‐positive fibres, whereas SERCA2‐positive fibre CSA was preserved. Notably, the radial atrophy of SERCA1 fibres was largely explained by a lower number of myofibrils, rather than a smaller size of the myofibrils. Similar alterations were observed in aged mice, although SERCA1 fibres also exhibited slightly smaller myofibril size. In mice, disuse likewise caused radial muscle fibre atrophy that was again almost exclusively associated with a lower number of myofibrils. Collectively, these findings identify the loss of myofibrils as a central and conserved mechanism that mediates radial muscle fibre atrophy during ageing and disuse, highlighting a potential therapeutic target for preserving skeletal muscle mass.

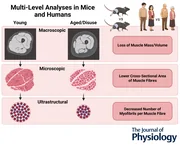

**Key points:**

- Skeletal muscles atrophy with ageing and disuse, and the loss of muscle mass increases the risk of falls, disability and all‐cause mortality.
- Skeletal muscles are composed of long cells called muscle fibres, and each fibre is densely packed with thread‐like structures called myofibrils; however, whether muscle fibre atrophy is primarily driven by reductions in the size of the myofibrils, and/or the loss of myofibrils, is not known.
- Using a next‐generation imaging pipeline, this study examined muscle samples from young and older adults, as well as from young and old mice.
- The results show that muscle fibre atrophy during both ageing and disuse is mainly associated with a lower number of myofibrils, rather than a smaller size of the myofibrils.
- Identifying the loss of myofibrils as the primary mechanism underlying muscle fibre atrophy highlights a specific therapeutic target for preserving muscle as people age or recover from injury.

## Graphical abstract

**Abstract figure legend** Skeletal muscle structure was analysed at three levels in young *vs*. aged humans, and in young *vs*. aged mice subjected to unilateral hindlimb immobilization to model disuse. (1) Macroscopic: magnetic resonance imaging of the thigh (human) and wet mass of individual hindlimb muscles (mouse) revealed that both ageing and disuse induce the loss of muscle mass/volume. (2) Microscopic: immunohistochemistry showed a decrease in the cross‐sectional area (i.e. radial atrophy) of Type II (SERCA1‐positive) fibres with both ageing and disuse, whereas Type I (SERCA2‐positive) fibres were preserved with ageing. (3) Ultrastructural: fluorescence imaging of myofibrils and image deconvolution (FIM‐ID), a next‐generation imaging pipeline, showed that the radial atrophy of the fibres was almost exclusively as a result of the decrease in the number of myofibrils per fibre, with minimal change in myofibril size. Collectively, these findings identify the loss of myofibrils as a central and conserved mechanism of muscle fibre atrophy.

## Introduction

Skeletal muscle is the largest internal organ in the human body, accounting for ∼35% of the total body mass (Janssen et al., 2000). Beyond its primary role in locomotion, skeletal muscle performs a variety of other functions, including thermoregulation, support of respiration, maintenance of metabolic homeostasis and endocrine signalling through the secretion of myokines (McLeod et al., 2016; Severinsen & Pedersen, 2020; Thyfault & Bergouignan, 2020). Given these multifaceted functions, it should not be surprising that the maintenance of skeletal muscle mass has become widely recognized as being critical for overall health and quality of life (Hanna et al., 2022; McLeod et al., 2016; Wolfe, 2006). Indeed, the loss of skeletal muscle mass has been associated with an increased risk of type II diabetes, cardiovascular diseases and all‐cause mortality (Li et al., 2018; Sizoo et al., 2023; Tyrovolas et al., 2020). Thus, understanding the mechanisms that regulate skeletal muscle mass is crucial, as this knowledge will guide the development of therapies for preventing the loss of muscle mass and improving public health.

At the most fundamental level, changes in skeletal muscle mass arise from alterations in the size and/or number of its structural components. These components are organized in a hierarchical manner that can be examined at three levels: the macroscopic level (visible without magnification), the microscopic level (observable using standard light microscopy) and the ultrastructural level (requiring high‐resolution imaging techniques such as electron microscopy or super‐resolution light microscopy) (Jorgenson et al., 2020; Sayed et al., 2023). Generally speaking, the macroscopic level encompasses the anatomical properties of the whole muscle, the microscopic level reflects features of the individual muscle fibres and the ultrastructural level pertains to the properties of the myofibrils that are found within the fibres. Importantly, the hierarchical organization of the structures predicts that adaptations at lower levels propagate upward in a predictable manner. For example, changes at the level of the myofibrils should lead to predictable changes at the level of the muscle fibres, which, in turn, should lead to predictable changes at the whole muscle level.

One of the most clinically relevant contexts for studying the mechanisms that regulate muscle mass is ageing. Specifically, with advancing age, skeletal muscle undergoes a progressive loss in mass through a process known as sarcopenia (Gustafsson & Ulfhake, 2024; Larsson et al., 2018). At the macroscopic level, numerous studies have demonstrated that sarcopenia is marked by a decrease in whole muscle cross‐sectional area (CSA), and that this effect has been attributed to co‐ordinated changes at the microscopic level, including both the loss of muscle fibres (i.e. hypoplasia) and a reduction in the CSA of the muscle fibres (i.e. radial atrophy) (Lexell et al., 1988; Nilwik et al., 2013).

Disuse represents another clinically relevant context in which skeletal muscle mass is profoundly reduced (Cuyul‐Vásquez et al., 2025; Nunes et al., 2022). Indeed, a variety of models in humans and lower organisms have consistently shown that disuse leads to a decrease in whole muscle CSA (Franchi et al., 2022; Sandona et al., 2012; Sayed et al., 2023). However, unlike with ageing, the disuse‐induced decline in whole muscle CSA does not appear to involve hypoplasia. Instead, most studies indicate that the decrease in whole muscle CSA is largely driven by radial atrophy of existing muscle fibres (Arentson‐Lantz et al., 2016; Mortreux et al., 2021; Sayed et al., 2023).

Although the induction of radial atrophy that occurs with disuse and ageing has been recognized since the 1970s, the underlying ultrastructural adaptations that drive this phenomenon remain poorly defined (Jaffe et al., 1978; Narici et al., 2016; Tomonaga, 1977). Indeed, even the most basic questions, such as whether the radial atrophy of the muscle fibres is driven by a decrease in the CSA of the myofibrils and/or a reduction in the number of myofibrils, remain unresolved. Thus, the primary objective of this study was to identify the macro‐to‐ultrastructural adaptations that mediate the ageing‐ and disuse‐induced loss of muscle mass.

## Methods

### Participants and ethical approval

Ten young males (19–40 years), eight young females (21–28 years), nine old males (69–84 years) and seven old females (65–72 years) volunteered and provided their written informed consent to participate in this study. Participants underwent a general health screening that included an assessment of body composition and thigh lean mass with dual X‐ray absorptiometry (Lunar iDXA; GE, Madison, WI, USA). Participants were excluded from the study if they were taking medications that may affect muscle mass (e.g. hormone‐replacement therapies, glucocorticoids, etc.). All participants were apparently healthy, community‐dwelling adults free of any known neurological, musculoskeletal, or cardiovascular diseases. Following the general health screening, participants reported to the laboratory on two occasions: once for a muscle biopsy of the vastus lateralis (VL), and one to quantify the size of the quadriceps muscles with magnetic resonance imaging (MRI). The order of these visits varied according to scanner availability and participant scheduling. When the muscle biopsy preceded the MRI assessment, the MRI was performed at least 4 days after the biopsy. In those participants, the biopsy site was not visibly identifiable on the MRI images. The anthropometrics of the young and old participants are shown in Table 1. All experimental procedures were approved by the Marquette University Institutional Review Board (Protocol Number HR‐2945) and conformed to the principles in the *Declaration of Helsinki*.

**Table 1:** Characteristics of the human participants

	Age (years)	Height (cm)	Body mass (kg)	Body fat (%)	BMI	Femur length (cm)	Thigh lean mass (kg)
**Male young**	27.1 ± 6.8	181.3 ± 8.6	84.1 ± 15.0	22.4 ± 3.8	25.4 ± 2.8	39.7 ± 4.5	8.8 ± 2.0
**Male old**	74.3 ± 6.5	177.4 ± 7.0	83.4 ± 3.6	31.5 ± 5.5	26.6 ± 2.5	37.4 ± 1.8	6.3 ± 0.8
**Female young**	23.0 ± 2.3	163.1 ± 9.7	63.1 ± 10.0	30.1 ± 7.5	23.7 ± 3.0	34.9 ± 1.3	5.4 ± 0.9
**Female old**	69.0 ± 2.3	160.5 ± 5.6	65.0 ± 12.6	36.3 ± 6.9	25.1 ± 3.9	34.6 ± 1.5	4.5 ± 0.6
**Age main effect**	***P* < 0.0001**	*P* = 0.2489	*P* = 0.8733	***P* = 0.0010**	*P* = 0.2168	*P* = 0.1850	***P = *0.0007**
**Sex main effect**	***P* = 0.0146**	***P* < 0.0001**	***P* < 0.0001**	***P* = 0.0054**	*P* = 0.1309	***P* = 0.0006**	***P < *0.0001**
**Interaction**	*P* = 0.7369	*P* = 0.8157	*P* = 0.7450	*P = *0.5020	*P* = 0.8935	*P* = 0.3111	*P* = 0.0834

Note: All values are presented as the mean ± SD from *n* = 7–10 per group. The data for each of the variables were analysed with two‐way ANOVA, and the *P* values for the main effects and interactions are listed.

### MRI of the quadriceps muscles

To quantify the volume and largest CSA of the quadriceps muscles, participants laid supine in a 3.0 Tesla superconducting magnet (Signa Premier; GE, Madison, WI, USA). The scanner operated with a 3D dual‐echo, fat‐water Dixon pulse sequence in the axial plane, yielding 7 mm thick serial cross‐sections with the following parameters: TR = 8.28 ms, echo time (TE) = 2.97 ms, imaging frequency = 127.8 MHz, flip angle = 5°. Participants remained supine for at least 20 min prior to scanning to eliminate the effects of postural shifts in fluid volume on MRI measurements (Berg et al., 1993). Axial slices from the T1‐weighted water images were analysed with Medical Image Processing, Analysis and Visualization (MIPAV, version 11.2.0; National Institutes of Health Centre for Information Technology, Bethesda, MD, USA) beginning at the first slice where the patella was no longer visible and continuing through the slice corresponding to 80% of the total femur length. Femur length was measured as the distance between the first slice that includes the full tibial plateau and the last slice where the most proximal portion of the femoral head was still visible. For each slice, the quadriceps muscles (VL, vastus intermedius and rectus femoris) were manually outlined if visible, and the corresponding volumes were quantified based on the CSA and slice thickness. The volumes for each of the four muscles were summed across all slices to determine muscle‐specific volumes, and the total quadriceps volume were determined as the combined volume of all four muscles. For ease of comparison to prior ageing skeletal muscle literature, we also report the single 7 mm thick slice with the largest quadriceps and VL CSA.

### Collection and processing of human skeletal muscle biopsies

A muscle biopsy from the VL was obtained from each participant as described previously (Sundberg et al., 2018). Briefly, participants were instructed to refrain from strenuous exercise of the lower limbs for 48 h prior to the biopsy and arrive at the laboratory fasted and without consumption of caffeine for ≥8 h. The biopsy site was cleaned with 70% ethanol, sterilized with 10% povidone‐iodine and anaesthetized with 1% lidocaine HCl. A small incision, ∼1 cm in length, was made over the distal third of the muscle belly, and the 6‐mm Bergström biopsy needle was inserted under local suction to obtain the tissue sample. A muscle bundle was aligned with the fibres oriented longitudinally on a small notecard presoaked in 0.1 m phosphate buffer (PB), and the sample was submerged in 0.1 m PB for 30 s to minimize potential dehydration of the bundle before undergoing tissue fixation and cryoprotection. To preserve the ultrastructural integrity of the myofibrils, we extensively fixed the sample in 4% paraformaldehyde and subsequently cryoprotected it with 15–45% sucrose as described previously (Jorgenson et al., 2024). Specifically, muscle bundles were fixed in 4% paraformaldehyde in 0.1 m PB for 24 h (3 h at 20°C on an orbital shaker at 50 rpm and 21 h at 4°C on a nutating rocker), and then cryoprotected by incubating in 15% sucrose in 0.1 m PB for 6 h (4°C) followed by 45% sucrose in 0.1 m PB for an additional 18 h (4°C). The fixed and cryoprotected sample was then immersed in optimal cutting temperature (OCT) compound, frozen in liquid nitrogen‐chilled isopentane and stored at –80°C until sectioning.

### Animals

Experimental procedures were conducted on adult (4 months old, obtained from Jackson Laboratory, Bar Harbor, ME, USA) and aged (24 months old, obtained from the National Institute on Aging rodent colonies) male C57BL/6 mice. These animals were housed at 25°C under a 12:12 h light/dark photocycle with free access to standard rodent chow and water, but no additional provisions for exercise enrichment such as running wheels or climbing devices. After shipment to the University of Wisconsin‐Madison, the mice were allowed a 1 week acclimation period and, prior to immobilization, the mice were anaesthetized with 2–5% isoflurane in oxygen. For tissue collection, animals were killed by cervical dislocation under anaesthesia, followed by removal of the hindlimbs. All procedures were approved by the Institutional Animal Care and Use Committee (IACUC) of the University of Wisconsin‐Madison (protocol #V005375).

### Hindlimb immobilization

Unilateral hindlimb immobilization was performed as previously described (You et al., 2015). Briefly, mice were anaesthetized with 2–5% isoflurane in oxygen, and the right hindlimb was immobilized with the knee extended and the ankle plantar‐flexed for 10 days. The contralateral (left) hindlimb remained unrestrained and served as a control. Following the immobilization period, mice were anaesthetized and various tissues from the hindlimbs were collected as described below.

### Collection and processing of mouse skeletal muscles

Hindlimb collection and tissue processing were performed as previously described (Jorgenson et al., 2024). Briefly, mice were anaesthetized with 2–5% isoflurane in oxygen, and then the skin on the hindlimbs was retracted and the hindlimbs were severed at the hip joint. Next, for each hindlimb, the foot and proximal femur were secured to a circular aluminum wire mesh with 90° angles in the ankle and knee joints using non‐absorbable silk sutures, and then they were fixed in 20 mL of 4% paraformaldehyde in 0.1 m PB for 3 h at room temperature on an orbital shaker at 50 rpm. Various muscles and the tibia from each hindlimb were then dissected, the length of the tibias and mass of the muscles were recorded, and then the soleus and flexor digitorum longus muscles were transferred to 1.5 mL tubes containing 1.0 mL of 4% paraformaldehyde in 0.1 m PB, and post‐fixed for 21 h at 4°C on a nutating rocker. The muscles were then cryoprotected and frozen in OCT as described for the human samples.

### Immunohistochemistry

Mid‐belly (mouse) and biopsy (human) muscle cross‐sections (5 µm thick) were obtained from OCT‐embedded tissue using a cryostat set to –30°C and mounted on Superfrost Plus microscope slides (Thermo Fisher Scientific, Waltham, MA, USA). Sections were immediately hydrated in diH_2_O for 15 min. At the same time as maintaining hydration, the area surrounding each section was gently dried with a Kimwipe (Kimberly‐Clark, Irvine, TX, USA), and a hydrophobic barrier was drawn around the tissue using a PAP pen (Aqua Hold 2; Scientific Device Laboratory, Des Plains IL, USA). Slides were then placed into a humidified chamber, where all subsequent washing and incubation steps were performed at room temperature on an orbital shaker at 50 rpm.

Sections were washed in phosphate‐buffered saline (PBS) for 5 min and then incubated for 30 min in blocking solution (0.5% Triton X‐100, 0.5% bovine serum albumin in PBS). For mouse samples, sections were incubated overnight with mouse IgG1 anti‐SERCA1 (dilution 1:100; VE121G9; Santa Cruz Biotechnology, Santa Cruz, CA, USA; #SC‐58287) and rabbit anti‐dystrophin (dilution 1:100; Thermo Fisher Scientific; #PA1‐21011) diluted in blocking solution. For human biopsy samples, sections were incubated overnight with either mouse IgG1 anti‐SERCA1 or mouse IgG1 anti‐SERCA2 (dilution 1:200; IID8; Santa Cruz Biotechnology; #SC‐53010) along with rabbit anti‐dystrophin. After the overnight incubation, sections were washed three times for 10 min in PBS, followed by three additional washes for 30 min in PBS. The sections were then incubated overnight in blocking solution containing Alexa Fluor 594‐conjugated goat anti‐mouse IgG (Fcγ Subclass 1 specific; dilution 1:2000; Jackson ImmunoResearch, West Grove, PA, USA; #115‐585‐205) and Alexa Fluor 488‐conjugated goat anti‐rabbit IgG (dilution 1:5000; Invitrogen, Carlsbad, CA, USA; #A11008). After incubation, the sections were washed three times for 10 min in PBS, followed by three additional washes for 30 min in PBS. The slides were then mounted with 4 µL of ProLong™ Gold Antifade Mountant (Thermo Fisher Scientific; #P36930) and covered with a No. 1 glass coverslip (Globe Scientific, Mahwah, NJ, USA; #1404‐10). Mounted sections were cured in the dark for at least 24 h before imaging.

### Assessment of whole muscle size and muscle fibre size

Human and mouse cross‐sections labelled for dystrophin and SERCA1/2 were imaged using a BZX700 automated inverted epifluorescence microscope (Keyence, Osaka, Japan) with a 10× objective lens. For each fluorescent channel (FITC and TxRED), image fields were acquired as either 3 × 3 (mouse soleus samples) or 5 × 5 (mouse flexor digitorum longus and human samples) tiles and stitched using Analyzer Software (Keyence). Whole muscle CSA in mice and the mean CSA of the fibres in both human and mouse samples were quantified using our previously published CellProfiler pipeline (Zhu et al., 2021). The results from these analyses are presented in the Appendix (Fig. A1).

### Fluorescence imaging of myofibrils and image deconvolution (FIM‐ID)

FIM‐ID was performed following our previously described method (Jorgenson et al., 2024). In brief, randomly selected regions of interest from cross‐sections labelled for dystrophin and SERCA1/2 were imaged using a THUNDER Imager Tissue 3D microscope (Leica, Wetzlar, Germany) with an HC PL APO 63×/1.40 oil immersion objective. Z‐stacks were acquired across the full sample thickness and processed using Leica's Small Volume Computational Clearing (SVCC) algorithm for deconvolution. The deconvolution images were then opened in ImageJ (NIH, Bethesda, MD, USA) and the Z‐plane with the most in‐focus image for SERCA1/2 was selected. The corresponding autofluorescence signal from the same plane was merged with the SERCA1/2 images using the Z‐Project function in ImageJ, and the resulting merged image was saved as a single greyscale TIFF image. The final merged images were converted to 16‐bit TIFF format, and the pixel density was standardized to 6144 × 6144 using the resize function in ImageJ.

### Automated measurements of myofibril size and number with FIM‐ID

For mouse samples, individual fibres were randomly selected from each FIM‐ID image by a blinded investigator and manually traced in ImageJ to measure fibre CSA, as previously described (Jorgenson et al., 2024). Preliminary analyses included 15–30 fibres per sample (15 fibres per soleus samples and 30 fibres per flexor digitorum longus samples), with additional fibres randomly selected until the average CSA of the randomly selected fibres was within ±10% of the mean fibre CSA obtained from the Keyence‐based analyses of the whole muscle section. Single‐fibre images of the signal for SERCA1 were then subjected to automated measurements of myofibril CSA with our ‘Myofibril CSA Analysis’ pipeline in CellProfiler, version 4.2.1 (Jorgenson et al., 2024). To estimate the number of myofibrils per fibre, each SERCA1 fibre image was processed with our ‘Intermyofibrillar Area’ pipeline in CellProfiler, which quantifies the area in each fibre that is occupied by intermyofibrillar components (e.g. sarcoplasmic reticulum and mitochondria). The intermyofibrillar area was then subtracted from the total fibre CSA to yield the myofibrillar area, which was then divided by the mean myofibril CSA to determine the number of myofibrils per fibre. In some instances, the analysed fibres were also grouped according to their oxidative capacity as previously described (Jorgenson et al., 2024).

For human samples, the same general method was used with two modifications. First, analyses were performed on images labelled for SERCA1 or SERCA2. Second, preliminary analyses included 30 fibres (15 SERCA1 and 15 SERCA2), with additional fibres randomly selected until the average CSA was within ±5% of the mean fibre CSA obtained from Keyence‐based analyses of the whole biopsy section.

### Assessment of tubular aggregates

Randomly selected FIM‐ID images of SERCA1 from young and old human VL, mouse soleus and mouse flexor digitorum longus muscles were assessed for the proportion of tubular aggregate‐positive fibres by blinded investigators. Briefly, for each FIM‐ID image, all SERCA1‐positive fibres that were in focus and entirely encompassed within the image were counted. Then, the number of these fibres that contained tubular aggregates was determined. Three FIM‐ID images per sample were analysed, and the total number of tubular aggregate‐positive and negative fibres from the three images was used to determine the proportion of tubular aggregate‐positive fibres for each sample (8–29 fibres per sample for the VL, 32–62 fibres per sample for the soleus and 49–76 fibres per sample for the flexor digitorum longus).

### Statistical analysis

As previously described, values that were more than three SDs from the mean within a given group were excluded as outliers (Jorgenson et al., 2024). As specified in the figure legends, statistical significance was assessed using unpaired *t* tests, two‐way analysis of variance (ANOVA), mixed two‐way ANOVA or repeated‐measures two‐way ANOVA. For ANOVA, planned comparisons were performed with Tukey's least significant difference when the number of comparisons did not exceed *k* – 1, where k is the number of groups (Keppel & Wickens, 2004) and Sidak's was used when *k* – 1 was exceeded (i.e. Fig. 7). *P < *0.05 was considered statistically significant. All analyses were conducted using Prism, version 11.0.2 (GraphPad Software Inc., San Diego, CA, USA).

## Results

### Ageing‐induced changes in the macroscopic structure of human skeletal muscle

To identify ageing‐induced changes in the macroscopic structure of human skeletal muscle, MRI images of the thigh were collected from young and old participants (Table 1). The MRI images were then used to determine the volume of the quadriceps muscles (i.e. the vastus medialis, VL, vastus intermedius and rectus femoris) and the largest CSA along the length of these muscles. As shown in Fig. 1, the results indicated that ageing was associated with a 34% lower quadriceps muscle volume and the largest CSA of the muscles was 32% smaller. Similar results were found when the measurements were focused specifically on the VL. Moreover, the outcomes revealed that the effects of ageing were not significantly influenced by the sex of the participants (Fig. 2).

**Figure 1:**
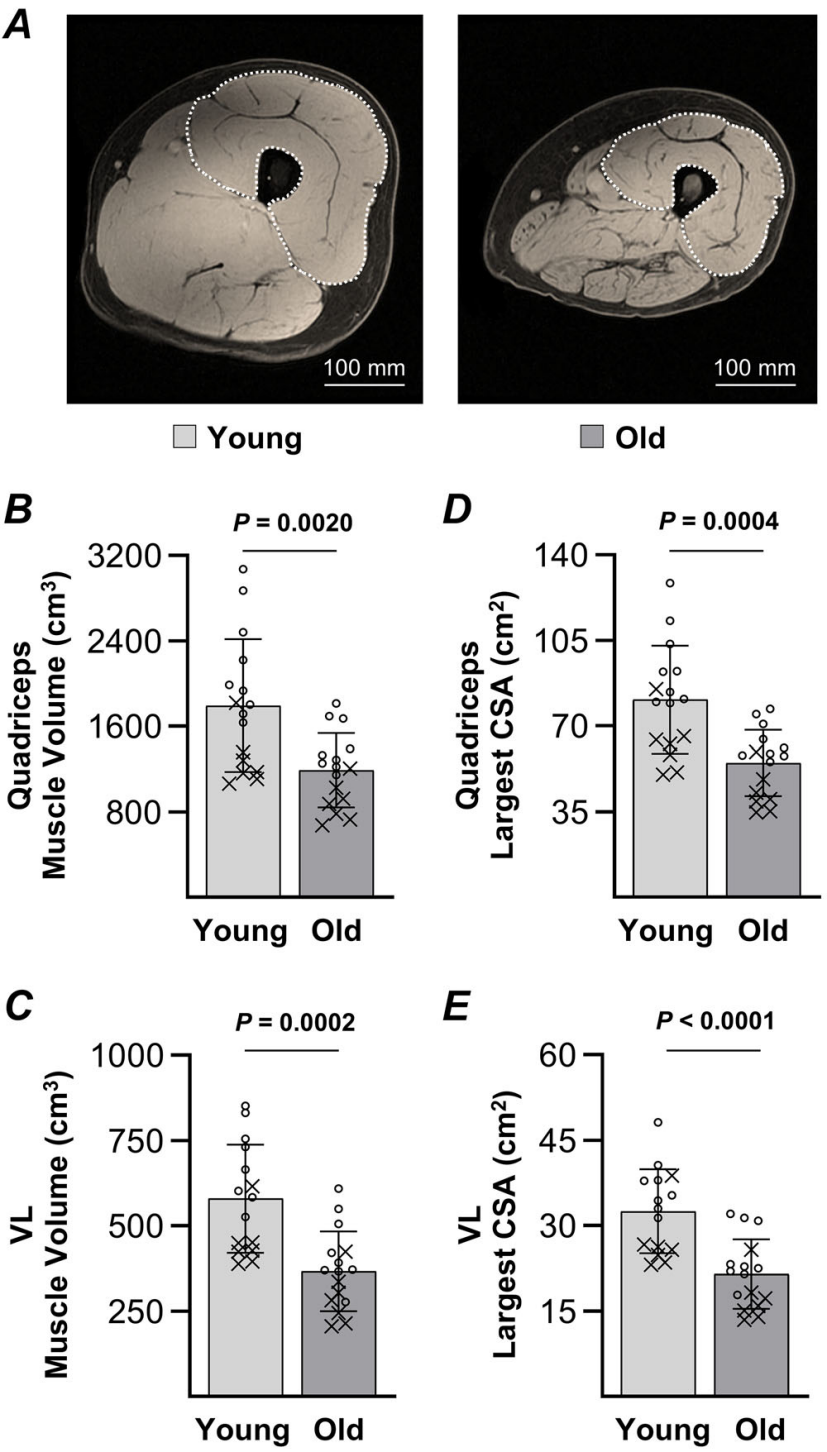
Ageing‐induced changes in the macroscopic structure of human skeletal muscle *A*, MRI images of the thigh were collected from young and old participants and used to determine (*B*) the volume of the quadriceps (highlighted with the white dashed line) and (*C*) vastus lateralis (VL), as well as the largest cross‐sectional area (CSA) along the length of the (*D*) quadriceps, and (*E*) VL. The images in (*A*) are from male participants, scale bars = 100 mm. The data in (*B*) to (*E*) represent the combined results from male (°) and female (X) participants. Values are presented as the group mean ± SD, *n* = 15–16 per group. The data were analysed with unpaired *t* tests, and the resulting *P* values are displayed in the graphs.

**Figure 2:**
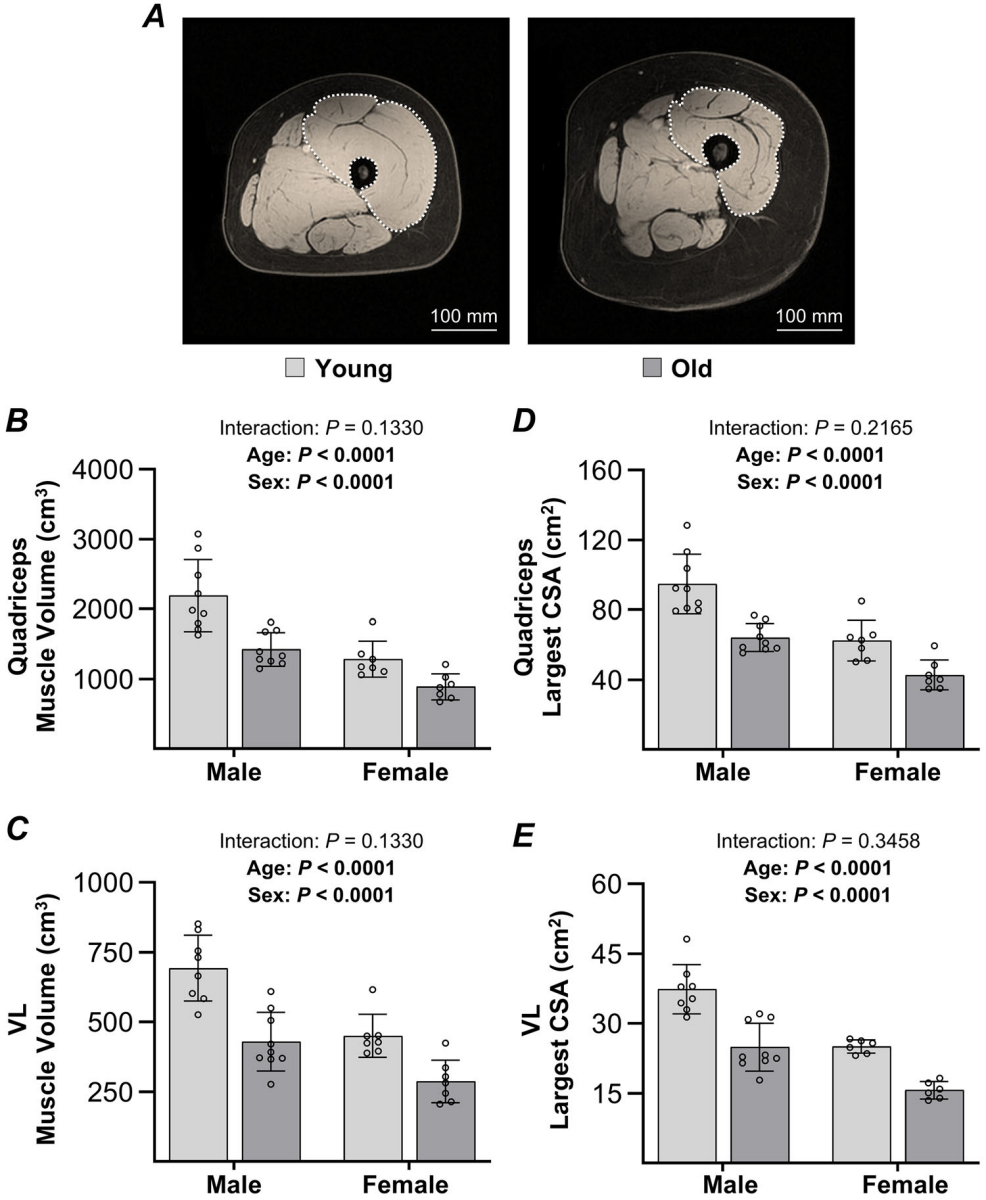
Ageing‐induced changes in the macroscopic structure of human skeletal muscle are not influenced by sex *A*, MRI images of the thigh were collected from young and old participants and used to determine (*B*) the volume of the quadriceps (highlighted with the white dashed line) and (*C*) vastus lateralis (VL), as well as the largest cross‐sectional area (CSA) along the length of the (*D*) quadriceps, and (*E*) VL. The images in (*A*) are from female participants, scale bars = 100 mm. The data in (*B*) and (*C*) were grouped by the age and sex of the participants and presented as the group mean ± SD, with *n* = 7–9 per group. The data were analysed with two‐way ANOVA, and the resulting *P* values for the main effects and interactions are displayed in the graphs.

### Ageing‐induced changes in the microscopic and ultrastructural properties of human skeletal muscle

Having established that ageing led to a significant decrease in the volume and CSA of the quadriceps muscles, we next sought to identify the microscopic and ultrastructural adaptations that mediated these effects. To accomplish this, biopsies from the VL of the participants were collected, and then cross‐sections were subjected to immunohistochemistry for dystrophin to identify the periphery of the muscle fibres, and for SERCA1 or SERCA2 to identify the periphery of the myofibrils in what generally corresponds with fast Type II fibres and slow Type I fibres, respectively (Fig. 3*A* and *B*) (Ahmed et al., 2019; Jorgenson et al., 2024). With respect to microscopic adaptations, the outcomes revealed that the effect of ageing was dependent on fibre type. Specifically, ageing led to a 23% decrease in the CSA of the SERCA1‐positive fibres (i.e. radial atrophy), but it did not significantly alter the CSA of the SERCA2‐positive fibres (Fig. 3*C*).

**Figure 3:**
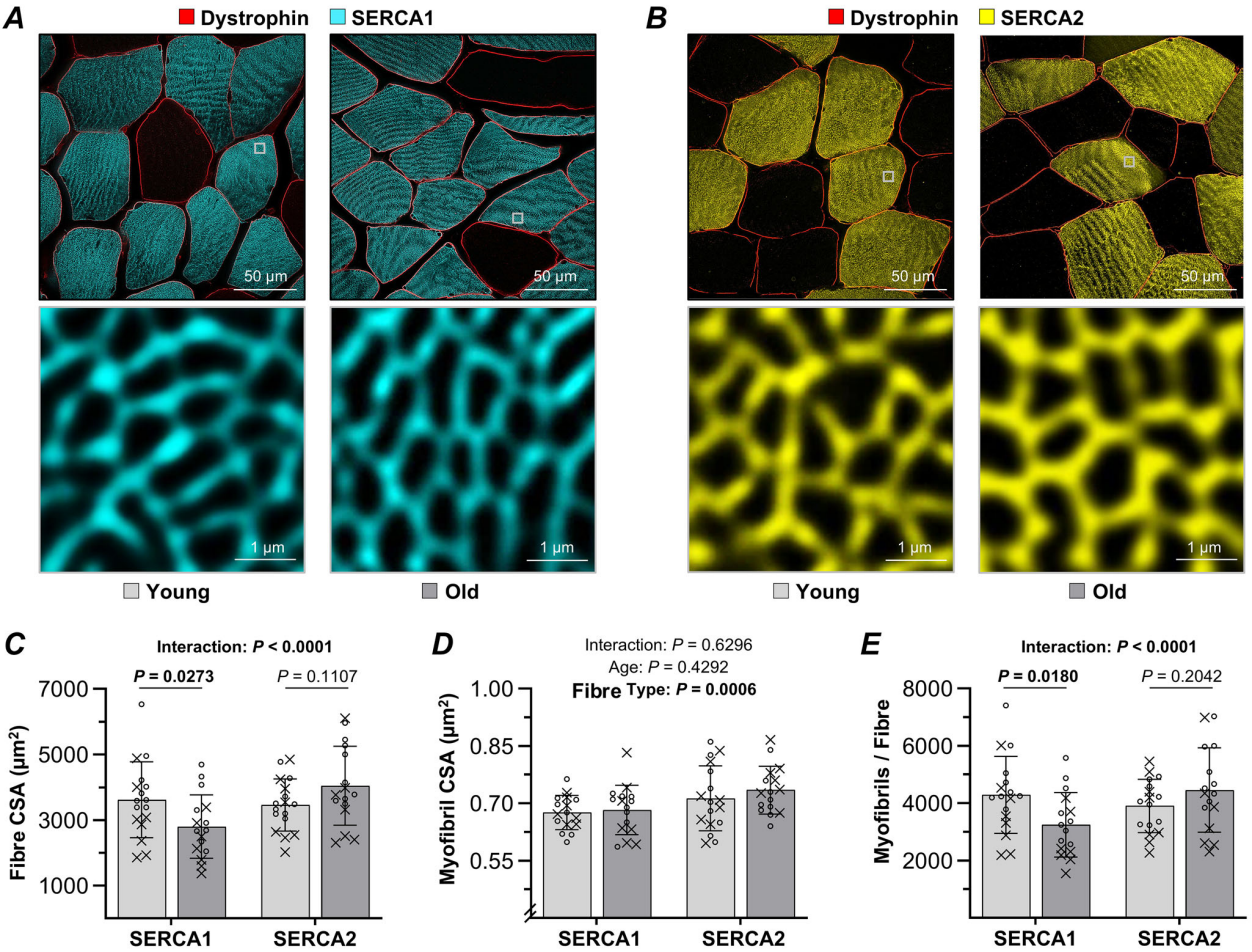
Ageing‐induced changes in the microscopic and ultrastructural properties of human skeletal muscle Biopsies of the vastus lateralis of young and old participants were collected and then cross‐sections were subjected to immunohistochemistry for dystrophin (to identify the periphery of muscle fibres) and (*A*) SERCA1 (to identify the periphery of the myofibrils in Type II fibres) or (*B*) SERCA2 (to identify the periphery of the myofibrils in Type I fibres). Scale bars = 50 µm in the low magnification images and 1 µm in the higher magnification images of the boxed regions. The images were used to determine the average (*C*) cross‐sectional area (CSA) of the fibres, (*D*) CSA of the myofibrils and (*E*) number of myofibrils per fibre. The images in (*A*) and (*B*) are from male participants, whereas the values in (*C*) to (*E*) represent the combined results from males (°) and females (X). Values are presented as the group mean ± SD, *n* = 16–17 independent muscles per group, 30–36 fibres per muscle, and an average of 957–1113 myofibrils per fibre. The data were analysed with repeated measures (*C* and *E*) or mixed (*D*) two‐way ANOVA, and the resulting *P* values for the main effects, interactions and pairwise comparisons are displayed in the graphs.

After determining that ageing led to radial atrophy of the SERCA1‐positive fibres, we sought to gain further insight into the ultrastructural adaptations that drove this effect. To do so, we used our recently developed FIM‐ID pipeline to measure the average CSA of the myofibrils, as well as the average number of myofibrils per fibre (Jorgenson et al., 2024). Notably, we previously benchmarked FIM‐ID against manual electron microscopy‐based measurements in mouse skeletal muscle and demonstrated that the two approaches yield highly concordant results (Jorgenson et al., 2024). As shown in Fig. 3*D*, the outcomes revealed that ageing did not significantly alter the CSA of the myofibrils in SERCA1‐ or SERCA2‐positive fibres. However, ageing was associated with a significant lower number of myofibrils per fibre, and just like the changes in fibre CSA, this effect was specific to the SERCA1‐positive fibres (Fig. 3*E*). As shown in Fig. 4, it was also determined that the ageing‐induced changes in the microscopic and ultrastructural properties of the SERCA1‐positive fibres were not significantly influenced by the sex of the participants.

**Figure 4:**
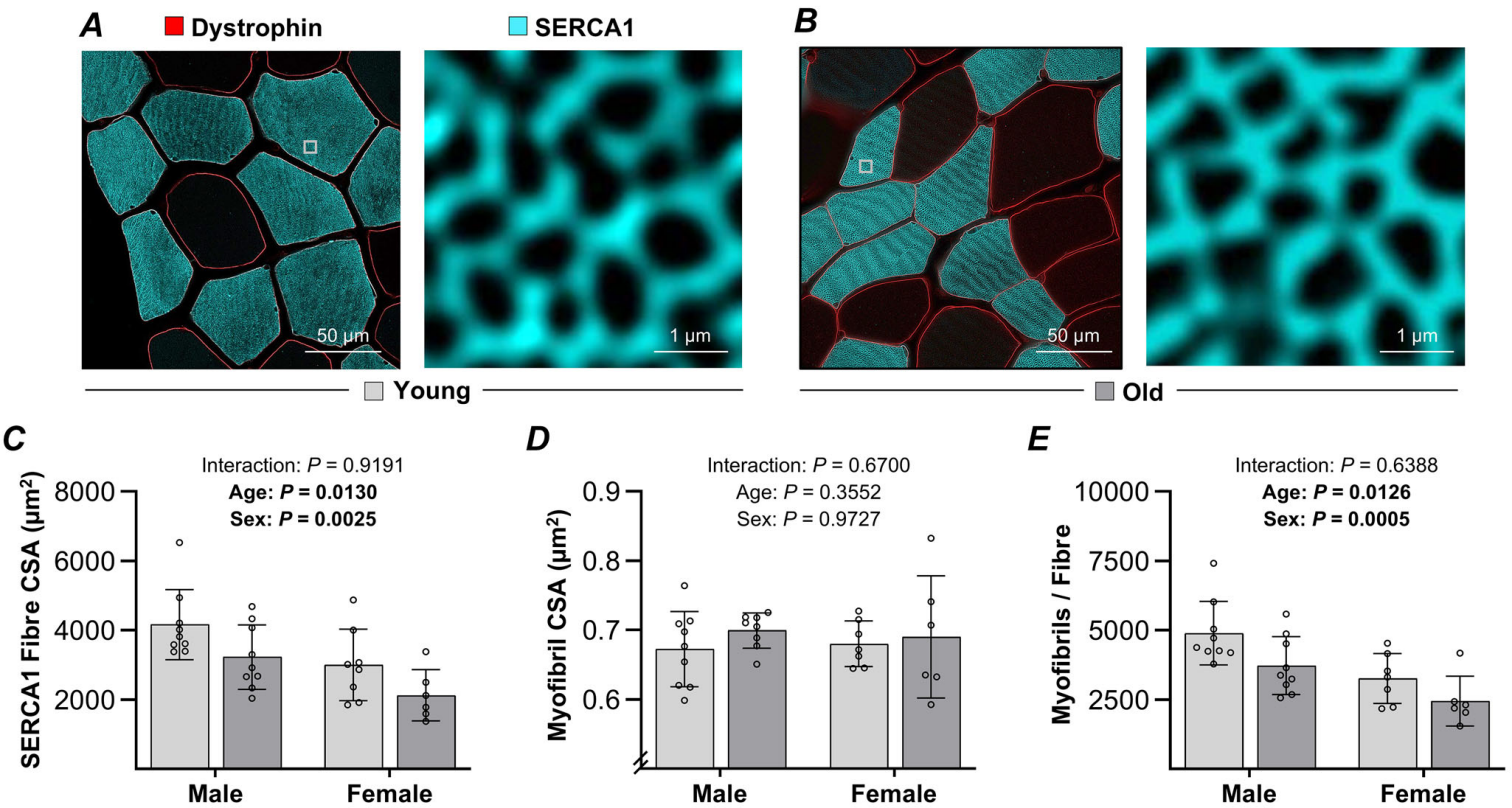
Ageing‐induced changes in the microscopic and ultrastructural properties of SERCA1‐positive fibres are not influenced by sex *A* and *B*, biopsies of the vastus lateralis of young and old participants were collected, and then cross‐sections were subjected to immunohistochemistry for dystrophin (to identify the periphery of muscle fibres) and SERCA1 (to identify the periphery of the myofibrils). Scale bars = 50 µm in the low magnification images and 1 µm in the higher magnification images of the boxed regions. The images were used to determine the average (*C*) cross‐sectional area (CSA) of the SERCA1‐positive fibres, (*D*) CSA of the myofibrils and (*E*) number of myofibrils per fibre. The data were grouped by the age and sex of the participants and presented as the group mean ± SD, *n* = 6–9 independent muscles per group, 15–18 fibres per muscle, and an average of 957–1113 myofibrils per fibre. The data were analysed with two‐way ANOVA, and the resulting *P* values for the main effects and interaction are displayed in the graphs. The images in (*A*) and (*B*) are from female participants.

### The effects of ageing and disuse on the mass of mouse skeletal muscles

Next, we wanted to examine whether the macro‐to‐ultrastructural changes observed with ageing in human skeletal muscle were conserved in mice. However, the use of aged mice is expensive, and therefore, we designed our experiments so that we could maximize their use. For example, the results in humans indicated that the effects of ageing were not significantly influenced by sex; thus, we focused our studies on a single sex, and we chose males because previous studies have reported that skeletal muscles of aged male mice more closely recapitulate the ageing phenotype of human skeletal muscle (de Jong et al., 2024). Furthermore, as illustrated in Fig. 5*A*, we subjected the young (4 months of age) and old (24 months of age) mice to a model of unilateral hindlimb immobilization, which allowed us to assess both the effects of ageing alone, as well as how ageing influences the loss of muscle mass that occurs in response to disuse. Specifically, at 10 days after the onset of the immobilization, body mass and the length of the tibia in each hindlimb were recorded (Fig. 5*B* and *C*), and then the tibialis anterior (TA), extensor digitorum longus (EDL), plantaris (PLT), soleus (SOL), gastrocnemius (GAST) and flexor digitorum longus (FDL) muscles from each hindlimb were weighed and saved for additional analyses (Fig. 5*D* and *E*).

**Figure 5:**
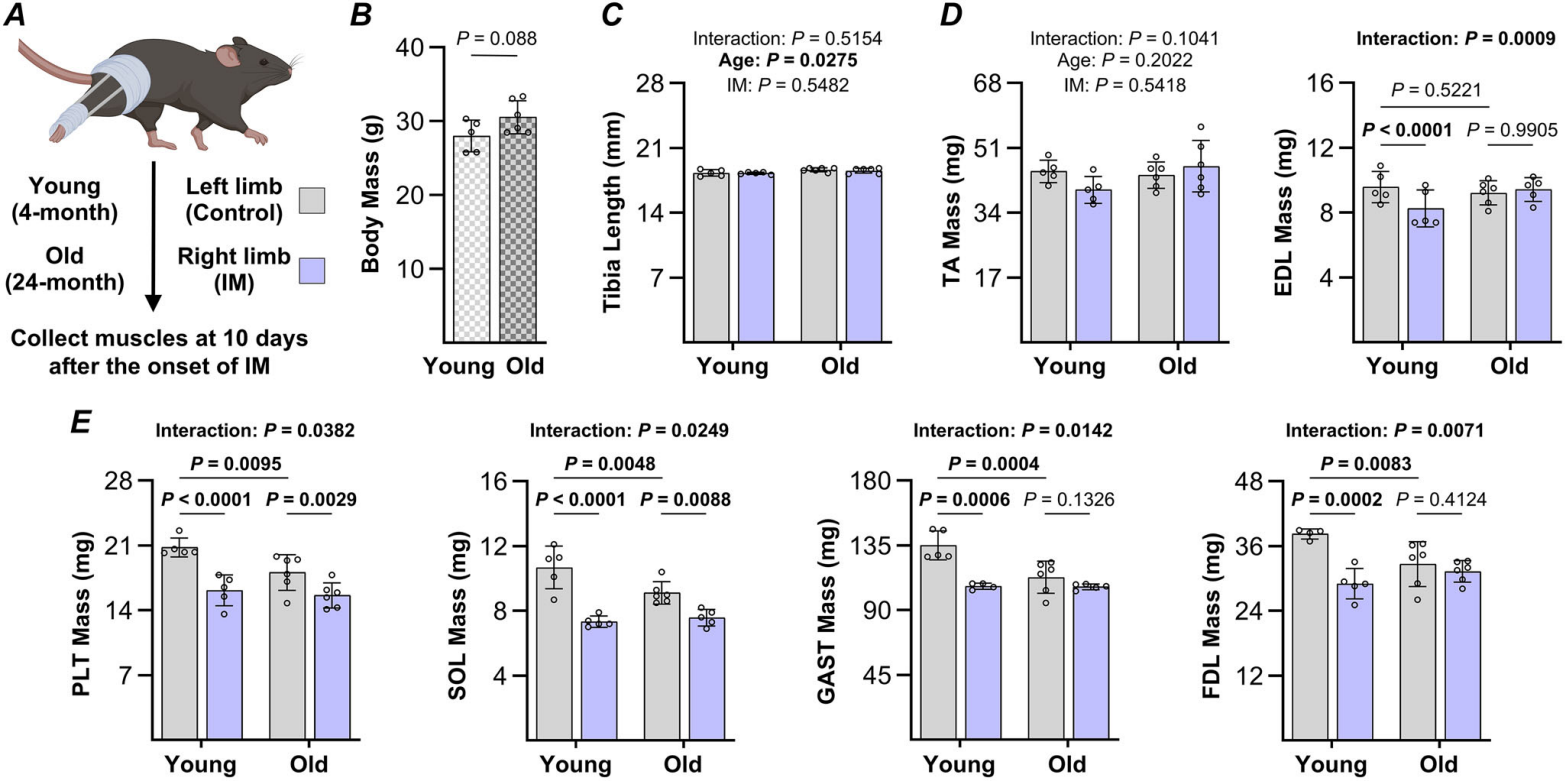
The effects of ageing and disuse on the mass of various mouse skeletal muscles *A*, young and old male C57BL6 mice were subjected to disuse via unilateral immobilization (IM) of the right hindlimb, whereas the left hindlimb served as a control (created with BioRender.com). After 10 days, measurements were obtained for body mass (*B*), tibia length (*C*) and the mass of individual hindlimb muscles, including (*D*) extensors such as the tibialis anterior (TA) and extensor digitorum longus (EDL), and (*E*) flexors such as the plantaris (PLT), soleus (SOL), gastrocnemius (GAST) and flexor digitorum longus (FDL). Values are presented as the group mean ± SD, *n* = 4–6 per group. The data were analysed with an unpaired *t* test (*B*), repeated measures two‐way ANOVA (*C* and *D*) or mixed two‐way ANOVA (*D* and *E*) and the resulting *P* values for the main effects, interactions and pairwise comparisons are displayed in the graphs.

To assess how immobilization and ageing impacted muscle mass, the data were first subjected to a three‐way ANOVA (i.e. age, immobilization and muscle), and the outcomes revealed that there was a significant interaction between the effects of age and immobilization, and that the interaction was dependent on the muscle being examined (*P* = 0.0003). Accordingly, we performed two‐way ANOVAs that assessed the effects of age and immobilization in each muscle, and the outcomes revealed that a significant interaction between these variables was present in all muscles except for the TA (Fig. 5*D* and *E*). Further analysis revealed that the interaction in these muscles could be attributed to a blunted immobilization‐induced decrease in mass in the aged animals. Subsequent pairwise analyses also revealed that all of the flexor muscles (i.e. the PLT, SOL, GAST and FDL) exhibited a significant ageing‐induced loss of mass, but this effect was not present in either of the extensor muscles (i.e. the TA and EDL).

### Ageing‐induced changes in the macro‐to‐ultrastructural properties of mouse skeletal muscle

Having established that various muscles of mice exhibit an ageing‐induced loss of mass, we next set out to determine whether this effect was mediated by the same macro‐to‐ultrastructural changes that we observed in humans. Specifically, as shown in Fig. 6, mid‐belly cross‐sections of the mixed slow/fast‐twitch SOL (i.e. Type I and IIa fibres) (Steinert et al., 2021), as well as the highly fast‐twitch FDL (i.e. Type IIa, IIx, and IIb fibres) (Zhu et al., 2021), were subjected to immunohistochemistry for dystrophin to identify the periphery of the muscle fibres, and SERCA1 to identify the periphery of the myofibrils in the Type II fibres (i.e. the fibre type that showed ageing‐induced radial atrophy in the human samples). Interestingly, during our analysis of the FDL muscles, we noticed non‐myofibrillar anomalies in the signal for SERCA1, with both the number and size of these anomalies being greater in the FDL muscles from the aged mice (Fig. 6*B*). We suspected that these anomalies represented tubular aggregates originally described by Engel et al. (1970) as an abnormal accumulation of the sarcoplasmic reticulum (Engel et al., 1970). As shown in Fig. 7, the presence of these anomalies also aligned with previous studies, which have shown that tubular aggregates are rare in healthy skeletal muscles of humans, but are prevalent in the fast‐twitch fibres of mouse skeletal muscles, where ageing leads to an increase in both their size and number (Agbulut et al., 2000; Engel et al., 1970). Importantly, because the presence of these anomalies would confound our assessment of myofibril CSA and myofibril number, all analyses for the FDL muscles were performed on fibres that did not contain the anomalies, yet were matched with the whole population in terms of their average CSA (for details, see Methods).

**Figure 6:**
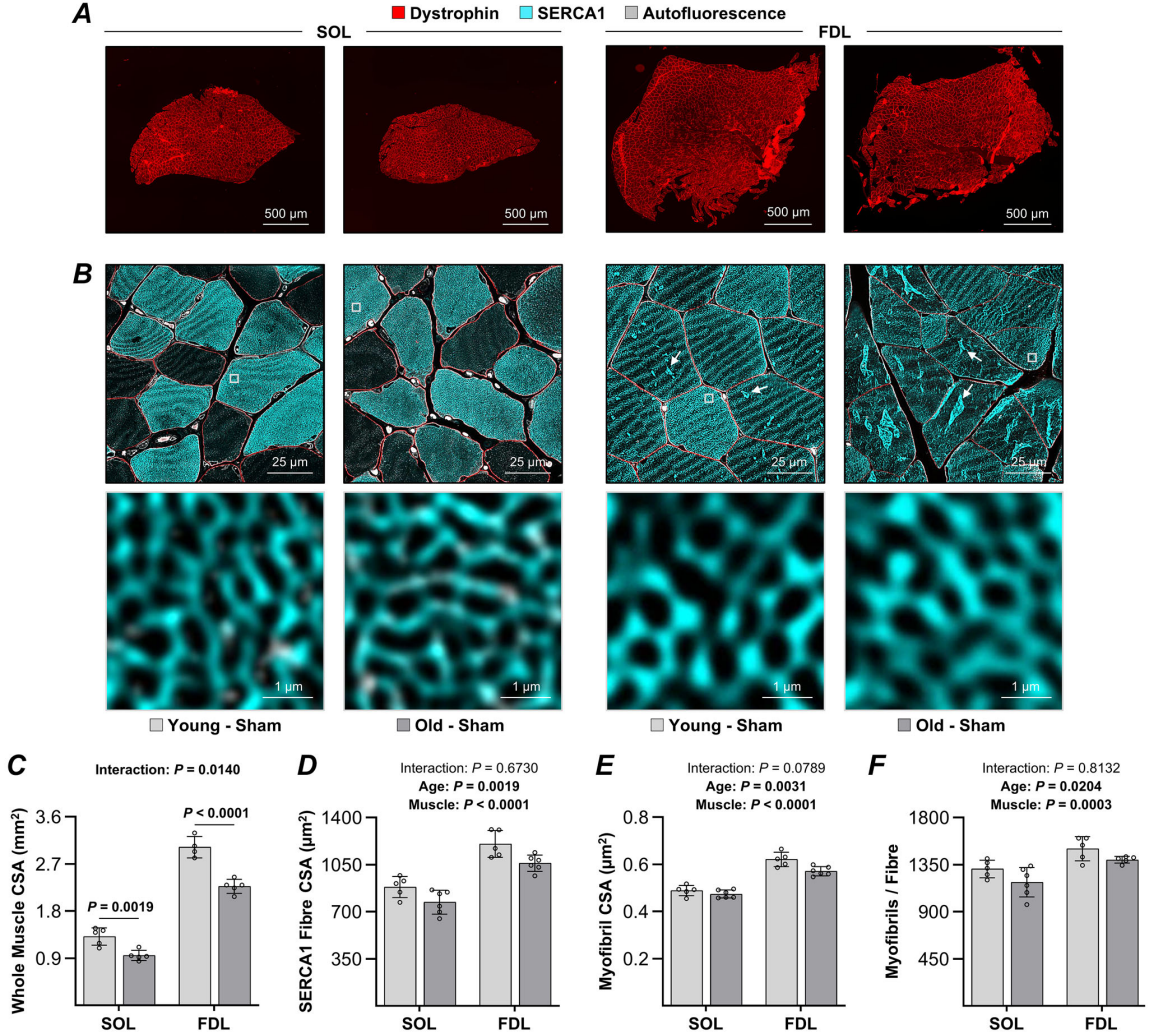
Ageing‐induced changes in the macro‐to‐ultrastructural properties of mouse skeletal muscle Mid‐belly cross‐sections were obtained from the SOL and FDL muscles of the control limb of the mice described in Fig. 3. The sections were subjected to immunohistochemistry for dystrophin (*A*), or dystrophin and SERCA1 (*B*), and imaged at various levels of magnification; scale bar in (*A*) = 500 µm, (*B*) = 25 µm in the low magnification images and 1 µm in the higher magnification images of the boxed regions. The resulting images were merged with the signal from autofluorescence and used to determine the (*C*) whole muscle cross‐sectional area (CSA), (*D*) the average CSA of the SERCA1‐positive fibres, as well as the (*E*) average CSA of the myofibrils, and (*F*) number of myofibrils per fibre in the SERCA1‐positive fibres. The data were grouped by the specific muscle and age, and presented as the group mean ± SD, *n* = 4–6 muscles per group, 15–34 fibres per muscle, and an average of 385–411 myofibrils per fibre. The data were analysed with two‐way ANOVA, and the resulting *P* values for the main effects, interactions and pairwise comparisons are displayed in the graphs. Arrows in (*B*) point to examples of the putative tubular aggregates.

**Figure 7:**
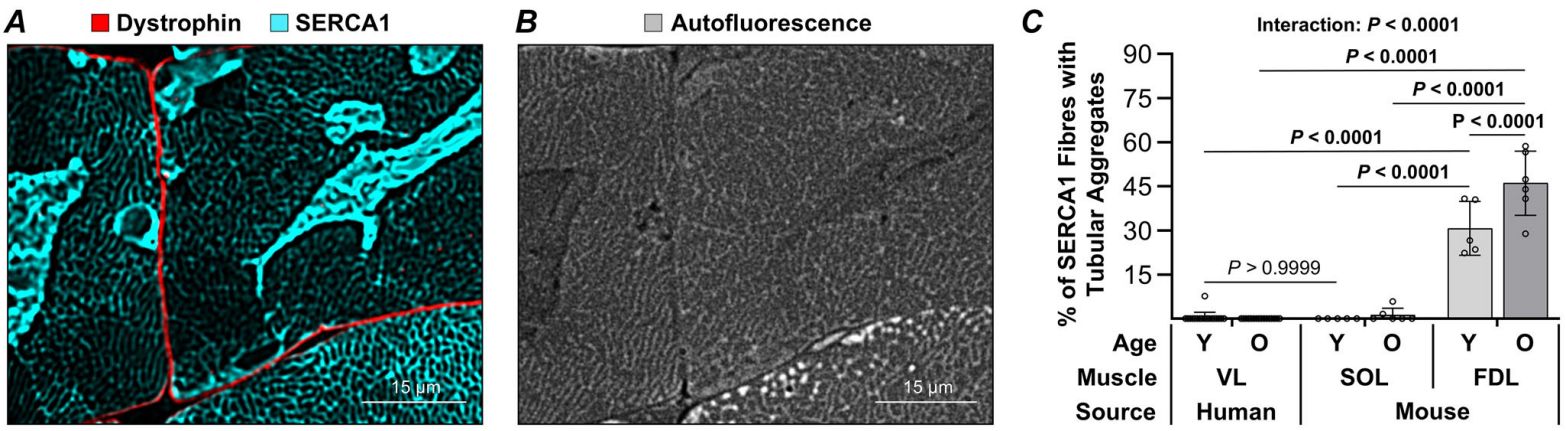
Ageing leads to an increase in tubular aggregates in the FDL muscle of mice *A*, representative image of the FDL from an old mouse that was subjected to immunohistochemistry for dystrophin (red) and SERCA1 (cyan), scale bar = 15 µm. *B*, an autofluorescent image of the same region shown in a reveals that the areas with intense SERCA1 staining (i.e. the tubular aggregates) exhibit a decrease in autofluorescence. *C*, images of SERCA1‐positive fibres in the vastus lateralis (VL) of young (Y) and old (O) humans (Fig. 2), as well as the images of SERCA1‐positive fibres from the control soleus (SOL) and control flexor digitorum longus (FDL) of Y and O mice (Fig. 4), were scored for the presence of tubular aggregates. Values are presented as the group mean ± SD, *n* = 4–6 per group. The data were analysed with two‐way ANOVA, and the resulting *P* values for the interaction and pairwise comparisons are displayed in the graphs.

As shown in Fig. 6*C*, the outcomes of our analyses revealed that the ageing‐induced loss of muscle mass in both the SOL and FDL was accompanied by a smaller whole muscle CSA. Similar to our observations in humans, we also found that ageing led to radial atrophy of the SERCA1‐positive fibres, and that this effect could be attributed, at least in part, to a lower number of myofibrils per fibre (Fig. 6*D*–*F*). However, unlike in humans, ageing also led to a small but significant decrease in the CSA of the myofibrils. Moreover, two‐way ANOVA revealed that there was a trend for this to occur in a muscle‐specific manner (3% *vs*. 8% in the SOL *vs*. FDL, respectively, *P* = 0.0789). Thus, although an ageing‐induced decrease in the number of myofibrils per fibre appears to be a conserved phenomenon, an ageing‐induced decrease in myofibril CSA might be an event that occurs specifically in mice and/or in a muscle‐/fibre type‐specific manner.

### The effect of ageing on disuse‐induced changes in the macro‐to‐ultrastructural properties of mouse skeletal muscle

After characterizing the macro‐to‐ultrastructural changes that are associated with the ageing‐induced loss of muscle mass, we then examined whether similar changes occur during the disuse‐induced loss of muscle mass and whether these effects are influenced by ageing. Specifically, mid‐belly cross‐sections were obtained from the SOL muscles of the control and immobilized limbs of both the young and old mice. The cross‐sections were then subjected to immunohistochemistry for dystrophin to identify the periphery of the muscle fibres and, for SERCA1, to identify the periphery of the myofibrils (Fig. 8*A* and *B*). In line with the changes that were observed for muscle mass (Fig. 5*E*), the outcomes revealed that immobilization led to a significant decrease in the whole muscle CSA of the SOL in young mice, and that the magnitude of this effect was significantly reduced in the SOL muscles from the old mice (Fig. 8*C*). It was also determined that immobilization led to radial atrophy of the SERCA1‐positive fibres and, just like the changes in whole muscle CSA, the magnitude of this effect was dependent on the age of the mice (Fig. 8*D*). Moreover, the radial atrophy of the SERCA1‐positive fibres could be attributed to a small immobilization‐induced decrease in the CSA of the individual myofibrils, along with a much more robust decrease in the number of myofibrils per fibre (Fig. 8*E* and *F*). It also bears mentioning that almost identical outcomes were observed when the same types of analyses were performed on FDL muscles (see Appendix, Fig. A2).

**Figure 8:**
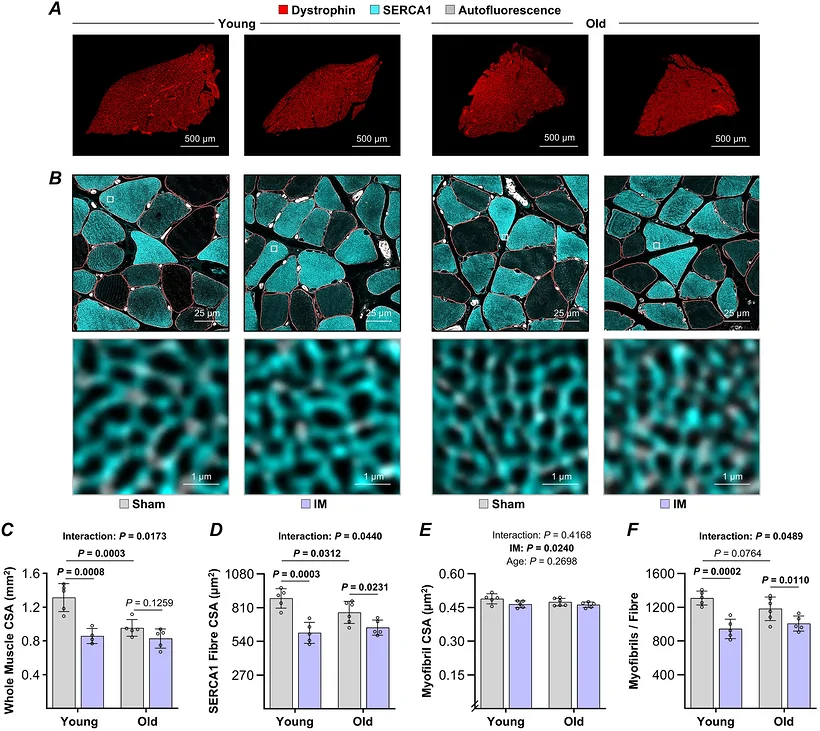
The effect of ageing on disuse‐induced changes in the macro‐to‐ultrastructural properties of the soleus muscle in mice Mid‐belly cross‐sections were obtained from the SOL muscles of the mice described in Fig. 3. The sections were subjected to immunohistochemistry for dystrophin (*A*), or dystrophin and SERCA1 (*B*), and imaged at various levels of magnification; scale bar (*A*) = 500 µm, (*B*) = 25 µm in the low magnification images and 1 µm in the higher magnification images of the boxed regions. The resulting images were merged with the signal from autofluorescence and used to determine the (*C*) whole muscle cross‐sectional area (CSA), (*D*) the average CSA of the SERCA1‐positive fibres, as well as the (*E*) average CSA of the myofibrils, and (*F*) number of myofibrils per fibre in the SERCA1‐positive fibres. The data were grouped by the specific muscle and age, and presented as the group mean ± SD, *n* = 4–6 independent muscles per group, 15–17 fibres per muscle, and an average of 369–399 myofibrils per fibre. The data were analysed with mixed two‐way ANOVA, and the resulting *P* values for the main effects, interactions and pairwise comparisons are displayed in the graphs.

## Discussion

For almost 50 years, studies have consistently concluded that the radial atrophy of muscle fibres is a major contributor to the loss of muscle mass that occurs during ageing and disuse (Arentson‐Lantz et al., 2016; Lexell et al., 1988; Mortreux et al., 2021; Nilwik et al., 2013). However, despite the widespread recognition of this phenomenon, the ultrastructural adaptations that drive the radial atrophy remain poorly defined. One reason for this gap is that myofibrils are the dominant ultrastructural component of the muscle fibres, and rigorous quantification of their properties requires the tracing of thousands of myofibrils per sample (Jorgenson et al., 2024). This challenge is further compounded by the fact that myofibrils are usually imaged with electron microscopy, a technique that generally does not provide the level of contrast needed for automated myofibril tracing (Jorgenson et al., 2024). As such, assessments of myofibril properties have traditionally relied on manual myofibril tracing (Jorgenson et al., 2024), and this means that even modestly powered studies could require hundreds to thousands of hours of labour. Thus, to address this limitation, we recently developed FIM‐ID, a light microscopy‐based approach that generates high‐contrast images that are amenable to automated myofibril quantification (Jorgenson et al., 2024).

With the advent of FIM‐ID, our group was uniquely equipped with the ability to assess the macro‐to‐ultrastructural adaptations that mediate the ageing‐ and disuse‐induced loss of skeletal muscle mass. In humans, the primary outcomes from our analyses revealed that ageing was associated with a decrease in quadriceps muscle volume and CSA that was accompanied by radial atrophy of SERCA1‐positive fibres. Importantly, our ultrastructural analyses revealed that the radial atrophy of the fibres was driven by a decrease in the number of myofibrils per fibre, with no change in the CSA of the individual myofibrils. This is important because it suggests that the ageing‐induced radial atrophy of the muscle fibres is mediated by a defect in myofibril turnover, rather than a more generalized and uniform degradation of the existing contractile machinery.

Parallel experiments in mice largely recapitulated the findings in humans, with co‐ordinated macro‐to‐ultrastructural declines occurring in multiple muscles during ageing. However, unique to mice, the CSA of the myofibrils also showed signs of a decrease, particularly in the highly fast‐twitch FDL muscle. The reason for this discrepancy is not known, but we suspect that it might at least partly be a result of differences in the fibre‐type composition of human and mouse skeletal muscles. Specifically, almost 60% of the fibres in the FDL are glycolytic/Type IIb (an isoform not present in human skeletal muscle), and we determined that the CSA of the myofibrils in oxidative SERCA1‐positive fibres of the FDL was not significantly altered by ageing, whereas the CSA of the myofibrils in the glycolytic SERCA1‐positive fibres was clearly lower (see Appendix, Fig. A3) (Harrison et al., 2011; Zhu et al., 2021). These results are consistent with the notion that the highly glycolytic/Type IIb fibres exhibit a distinct vulnerability to ageing. In further support of this, we also determined that tubular aggregates were highly prevalent in the FDL muscles of the old mice, but were rarely observed in the mixed slow/fast twitch SOL of the mice or the VL of the humans. Indeed, previous studies have reported that tubular aggregates are found exclusively within Type IIb fibres of aged mice (Agbulut et al., 2000). Thus, although our collective findings suggest that the overall trajectory of macro‐to‐ultrastructural adaptations that occur with ageing are broadly conserved, there are also unique ageing‐induced adaptations that occur in muscles with highly glycolytic/Type IIb fibres.

The analyses performed in the present study also provide insight into the mechanisms that mediate disuse‐induced muscle atrophy and how these processes are altered with advancing age. Specifically, immobilization resulted in radial atrophy of the muscle fibres, and this effect was associated with a lower CSA of the myofibrils and the number of myofibrils. These findings align with prior reports demonstrating rapid contractile protein loss during periods of inactivity (Brocca et al., 2015; Ferrando et al., 1996; Hinks & Power, 2024) and extend the literature by revealing that the structural basis for this loss involves remodelling at multiple ultrastructural levels. Notably, our analyses also revealed that the magnitude of disuse‐induced changes was markedly blunted in aged muscle. However, this was not entirely unexpected because previous studies have reported similar results (Baehr et al., 2017; Reidy et al., 2019) and might simply reflect the fact that aged mice exhibit a substantial decline in daily physical activity (Yanai & Endo, 2021). Put differently, we suspect that many of the muscles in the aged animals were conditioned with chronic inactivity/disuse and were therefore less responsive to the additional atrophic effects of immobilization.

Although the comparative nature of our study is a major strength, there are also several limitations that need to be acknowledged. For example, the analyses in humans were restricted to the VL and therefore would not capture muscle‐specific differences that probably exist across the body (Naruse et al., 2023). Second, the cross‐sectional nature of our studies precludes definitive conclusions about causality or the temporal sequence of the structural adaptations. Third, although the immobilization model enabled mechanistic insights into disuse‐induced atrophy, differences in fibre‐type composition between species (particularly the presence of highly glycolytic/Type IIb fibres in mice) limit the extrapolation of certain findings to humans. Finally, although our use of FIM‐ID provided robust ultrastructural measurements, it did not provide insight into the molecular mechanisms (e.g. signalling pathways, protein turnover rates, etc.) that drove the observed changes. Thus, additional studies will be needed to define these important mechanisms.

In conclusion, the present study provides a comprehensive macro‐to‐ultrastructural framework for understanding how ageing and disuse lead to the loss of skeletal muscle mass. The results indicate that a decrease in myofibril number represents a central and conserved mechanism underlying radial atrophy, whereas changes in myofibril size contribute in a context‐ and muscle‐/fibre type‐specific manner. These findings not only clarify long‐standing questions regarding the structural basis of muscle atrophy, but also highlight potential targets for therapeutic interventions aimed at preserving muscle mass and function across the lifespan. Future work should focus on identifying the molecular pathways that regulate myofibril turnover and determining how these processes can be modulated to enhance muscle resilience in ageing and disease.

## Additional information

## Competing interests

The authors declare that they have no competing interests.

## Author contributions

R.K.A.S., A.N.L., H.G.P., J.H., C.W.S and T.A.H were responsible for the conception or design of the work. R.K.A.S., A.N.L., H.G.P., J.E.H., M.M., C.G.K.F., J.H., M.B.Z.S., I.D., D.J.W., C.S.Z., J.J.J., C.W.S and T.A.H were responsible for the acquisition, analysis or interpretation of data for the work. R.K.A.S., A.N.L., H.G.P., J.E.H., M.M., C.G.K.F., J.H., M.B.Z.S., I.D., D.J.W., C.S.Z., J.J.J., C.W.S and T.A.H approved the final version of the manuscript submitted for publication. All authors qualify for authorship, and all those who qualify for authorship are listed. All authors also agree to be accountable for all aspects of the work in ensuring that questions related to the accuracy or integrity of any part of the work are appropriately investigated and resolved.

## Funding

This work was supported by the National Institute on Aging grant AG048262 to CWS and the National Institute of Arthritis and Musculoskeletal and Skin Diseases grants AR082816 and AR057347 to TAH, and AR085939 to CGKF. We thank Kam Kroner and Lanny Klamecki for assisting with a portion of the MRI analysis.

## Generative AI statement

Generative AI was used for editorial support and icon modification for the graphical abstract. Specifically, Claude Opus 4.8 was used to assess grammar, syntax and readability of the original author‐written text. Google Gemini 3.1 was used to modify icons that were derived from BioRender. No part of the study design, data analysis, interpretation or conclusions was generated by AI. The authors verified all content and accept full responsibility for the final manuscript.

## Supporting information


Peer Review History

## Figures and Tables

**Figure A1:**
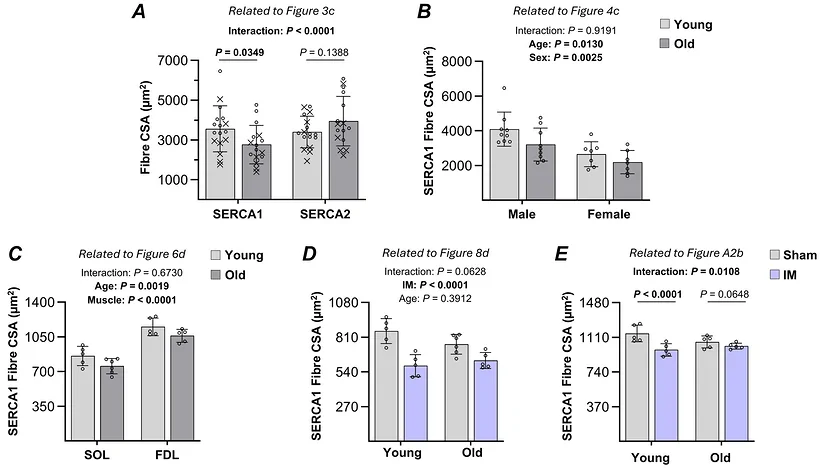
Keyence‐based cross‐sectional area of muscle fibres Muscle cross‐sections were subjected to immunohistochemistry for dystrophin (to identify the periphery of muscle fibres) as well as SERCA1 and/or SERCA2. Tiled images were stitched into high‐resolution images of the entire cross‐section using Keyence Analyzer Software and then processed with CellProfiler to determine the mean cross‐sectional area of the SERCA1 and/or SERCA2 fibres. *A* and *B*, vastus lateralis biopsies from young and old participants were grouped by age and fibre type (*A*; related to Fig. 3*C*; *n* = 16–17 independent muscles per group, 62–571 fibres per muscle), or by age and sex (*B*; related to Fig. 4*C*; *n* = 6–9 independent muscles per group, 67–452 fibres per muscle). *C*–*E*, mid‐belly cross‐sections of SOL and FDL muscles from the control limb of the mice described in Fig. 3 grouped by muscle and age (*C*; related to Fig. 6*D*; *n* = 5–6 muscles per group, 295–1143 fibres per muscle), by age and immobilization state for the SOL (*D*; related to Fig. 8*D*; *n* = 5–6 independent muscles per group, 295–588 fibres per muscle), and by age and immobilization state for the FDL (*E*; related to Fig. A2 *B*; *n* = 5 independent muscles per group, 660–1143 fibres per muscle). All *n* values are indicative of independent muscles. Data in (*A*) combine males (°) and females (X). Data are the group mean ± SD, analysed by repeated‐measures (*A*), standard (*B* and *C*) or mixed (*D* and *E*) two‐way ANOVA; *P* values for main effects, interactions and pairwise comparisons are displayed in the graphs.

**Figure A2:**
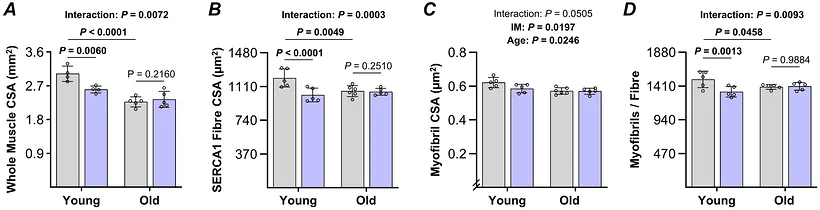
The effect of ageing on disuse‐induced changes in the macro‐to‐ultrastructural properties of the FDL muscle in mice Mid‐belly cross‐sections were obtained from the FDL muscles of the mice described in Fig. 3, processed as in Fig. 5, and used to determine the (*A*) whole muscle cross‐sectional area (CSA), (*B*) the average CSA of the SERCA1‐positive fibres, as well as the (*C*) average CSA of the myofibrils, and (*D*) number of myofibrils per fibre in the SERCA1‐positive fibres. The data were grouped by the specific muscle and age, and presented as the group mean ± SD, *n* = 4–6 independent muscles per group, 30–34 fibres per muscle, and an average of 368–411 myofibrils per fibre. The data were analysed with mixed two‐way ANOVA, and the resulting *P *values for the main effects, interactions and pairwise comparisons are displayed in the graphs.

**Figure A3:**
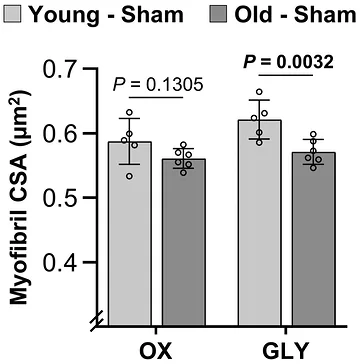
The effect of ageing on the CSA of myofibrils in the oxidative *vs*. glycolytic SERCA1‐positive fibres of the mouse FDL Mid‐belly cross‐sections of the FDL muscles were subjected to immunohistochemistry for dystrophin and SERCA1 and subsequently imaged as described in Fig. 4. The autofluorescence signal was used to classify the SERCA1‐positive fibres as being oxidative (OX) or glycolytic (GLY), and the mean CSA of the myofibrils for each fibre type was then determined. The resulting data were grouped by the specific fibre type and age, and presented as the group mean ± SD, *n* = 4–6 independent muscles per group, 30–34 fibres per muscle, and an average of 343–401 myofibrils per fibre. The data were analysed with unpaired *t* tests, and the resulting *P* values are displayed in the graphs.

## Data Availability

The authors confirm that the data supporting the findings of this study are available within the published article.
