# Supplementary material for: Macroscopic to ultrastructural analyses identify the loss of myofibrils as the primary mediator of ageing‐ and disuse‐induced muscle fibre atrophy
Source: J Physiol. 2026 Sep 25;604(20):8523–41. doi: 10.1113/JP291728 (PMC13674183; doi:10.1113/JP291728)
Supplement: Supplementary file 1 [file TJP-604-8523-s001.pdf]

The Journal of Physiology

Macroscopic to Ultrastructural Analyses Identify the Loss of Myofibrils as the Primary Mediator of Aging- and Disuse-Induced Muscle Fiber Atrophy

Ramy K. A. Sayed, Anthony N Lange, Hector G Paez, Jamie E. Hibbert, Marius Meinhold, Corey G.K. Flynn, Jared Hartung, Matilde B.Z. Vergara Santibanez, Isabell Dobrzycki, David J. Wrucke, Carlos S. Zepeda, Jessica J. James, Christopher W. Sundberg, and Troy A. Hornberger
DOI: 10.1113/JP291728

Corresponding author(s): Troy Hornberger (troy.hornberger@wisc.edu)

The following individual(s) involved in review of this submission have agreed to reveal their identity: Casper Soendenbroe (Referee #1)

Review Timeline:

Submission Date:	11-May-2026
Editorial Decision:	27-Jun-2026
Revision Received:	03-Aug-2026
Accepted:	17-Aug-2026

Senior Editor: Karyn Hamilton

Reviewing Editor: Martino Franchi

Transaction Report:

(Note: With the exception of the correction of typographical or spelling errors that could be a source of ambiguity, letters and reports are not edited. Depending on transfer agreements, referee reports obtained elsewhere may or may not be included in this compilation. Referee reports are anonymous unless the Referee chooses to sign their reports.)

Re: JP-RP-2026-291728 "**Macroscopic to Ultrastructural Analyses Identify the Loss of Myofibrils as the Primary Mediator of Aging- and Disuse-Induced Muscle Fiber Atrophy**" by Ramy K. A. Sayed, Anthony N Lange, Hector G Paez, Jamie E. Hibbert, Marius Meinhold, Corey G.K. Flynn, Matilde B.Z. Santibanez, Isabell Dobrzycki, David J. Wrucke, Carlos S. Zepeda, Jessica J. James, Christopher W. Sundberg, and Troy A. Hornberger

Dear Dr Hornberger,

Thank you for submitting your manuscript to The Journal of Physiology. It has been assessed by a Reviewing Editor and by 2 expert referees and we are pleased to tell you that it is potentially acceptable for publication following satisfactory major revision.

Please advise your co-authors of this decision as soon as possible.

The referee reports are copied at the end of this email.

Please address all the points raised and incorporate all requested revisions or explain in your Response to Referees why a change has not been made. We hope you will find the comments helpful and that you will be able to return your revised manuscript within 2 months. If your article is NOT for a Special Issue, you may have 9 months to revise. If you require an extension, please contact journal staff: jp@physoc.org. Please note that this letter does not constitute a guarantee for acceptance of your revised manuscript.

Your revised manuscript should be submitted online using the link in your Author Tasks: Link Not Available. This link is accessible via your account as Corresponding Author; it is not available to your co-authors. If this presents a problem, please contact journal staff (jp@physoc.org). Image files from the previous version are retained on the system. Please ensure you replace or remove any files that are being revised.

If you do not wish to submit a revised version of your manuscript, you must inform our journal staff (jp@physoc.org) or reply to this email to request withdrawal. Please note that a manuscript must be formally withdrawn from the peer review process at one journal before it may be submitted to another journal.

TRANSPARENT PEER REVIEW POLICY: To improve the transparency of its peer review process, The Journal of Physiology publishes online as supporting information the peer review history of all articles accepted for publication. Readers will have access to decision letters, including Editors' comments and referee reports, for each version of the manuscript, as well as any author responses to peer review comments. Referees can decide whether or not they wish to be named on the peer review history document.

ABSTRACT FIGURES: Authors are expected to use The Journal's premium BioRender account to create/redraw their Abstract Figures. Information on how to access this account is here: https://physoc.onlinelibrary.wiley.com/journal/14697793/biorender-access.

This will enable Authors to create and download high-resolution figures. If authors have used the free BioRender service, they can use the instructions provided in the link above to download a high-resolution version suitable for publication.

The link provided should only be used for the purposes of this submission. Authors will be charged for figures created on this account if they are not related to this manuscript submission.

LANGUAGE EDITING AND SUPPORT FOR PUBLICATION: If you would like help with English language editing, or other article preparation support, Wiley Editing Services offers expert help, including English Language Editing, as well as translation, manuscript formatting, and figure formatting at www.wileyauthors.com/eeo/preparation. You can also find resources for Preparing Your Article for general guidance about writing and preparing your manuscript at www.wileyauthors.com/eeo/prepresources.

RIGOUR AND REPRODUCIBILITY: We recommend authors consult The Journal's Rigour and Reproducibility requirements to ensure that their manuscript is suitable for publication: https://physoc.onlinelibrary.wiley.com/pb-assets/hub-assets/physoc/documents/TJP-Rigour-and-Reproducibility-Requirements.pdf

REVISION CHECKLIST:

Check that your Methods section conforms to journal policy: https://jp.msubmit.net/cgi-bin/main.plex?form_type=display_requirements#methods.

Check that data presented conforms to the statistics policy: https://jp.msubmit.net/cgi-bin/main.plex?form_type=display_requirements#statistics.

Upload a full Response to Referees file. To create your 'Response to Referees': copy all the reports, including any comments from the Senior and Reviewing Editors, into a Microsoft Word, or similar, file and respond to each point, using

font or background colour to distinguish comments and responses and upload as the required file type.

Please upload two versions of your manuscript text: one with all relevant changes highlighted and one clean version with no changes tracked. The manuscript file should include all tables and figure legends, but each figure/graph should be uploaded as separate, high-resolution files.

You may also upload:

- 'Potential Cover Art' for consideration as the issue's cover image
- Appropriate Supporting Information (Video, audio or data set: see https://jp.msubmit.net/cgi-bin/main.plex?form_type=display_requirements#supp).

We look forward to receiving your revised submission.

If you have any queries, please reply to this email and we will be pleased to advise.

Yours sincerely,

Karyn Hamilton
Senior Editor
The Journal of Physiology

REQUIRED ITEMS

- Author photo and profile. First or joint first authors are asked to provide a short biography (no more than 100 words for one author or 150 words in total for joint first authors) and a portrait photograph. These should be uploaded and clearly labelled together in a Word document with the revised version of the manuscript. See Information for Authors for further details.

- Your manuscript must include a complete Additional Information section, including competing interests; funding; author contributions and acknowledgements.

- Please upload separate high-quality figure files via the submission form.

- Please ensure that the Article File you upload is a Word file.

- Please include an Abstract Figure file and an Abstract Figure legend. An appropriate figure legend, which should not exceed 150 words in length, should be included in the main manuscript file. The Abstract Figure is a piece of artwork designed to give readers an immediate understanding of the research and should summarise the main conclusions. If possible, the image should be easily 'readable' from left to right or top to bottom. It should show the physiological relevance of the manuscript so readers can assess the importance and content of its findings. Abstract Figures should not merely recapitulate other figures in the manuscript. Please try to keep the diagram as simple as possible and without superfluous information that may distract from the main conclusion(s). Abstract Figures must be provided by authors no later than the revised manuscript stage and should be uploaded as a separate file during online submission labelled as File Type 'Abstract Figure'. Please also ensure that you include the figure legend in the main article file. All Abstract Figures should be created using BioRender. Authors should use The Journal's premium BioRender account to export high-resolution images. Details on how to use and access the premium account are included as part of this email.

- Please include a full title page as part of your main article (Word) file, which should contain the following: title, authors, affiliations, corresponding author name and contact details, keywords, and running title.

- Please ensure that all figures and tables have a title and legend, and that they have been cited within the main article text.

- Authors are required to fully disclose the use of any Generative AI (GAI) tools used in the preparation of their manuscript. This disclosure should also be included as a statement alongside the Additional Information and Data Availability statements towards the end of the article under the heading 'Generative AI Statement', and include the name(s), model and version of AI technology used, and exact details on the purpose for, and methods of, its use.

If no AI tool has been used, this should also be specifically stated within the manuscript.

EDITOR COMMENTS

Thank you for submitting your manuscript to the Journal of Physiology.

The manuscript has now been reviewed by two reviewers expert in the topic. Both reviewers (an myself as Reviewing Editor) found the study to be well executed and a likely important contribution to the field. Specifically, the application of high-resolution microscopy to quantify myofibrillar number was viewed likely to have broad impact.

In preparing your revision, I suggest you to carefully address the following key points raised by the reviewers:

1- clarification and validation of the myofibril quantification approach - Reviewer 1 requests a clearer description of how myofibril number is derived from image analysis, specifically whether it is calculated rather than directly counted. If so, please justify this approach

2- the methodological validation of the high-resolution imaging technique should be more explicitly discussed. While prior validation against electron microscopy is acknowledged, limitations-especially the lack of direct validation in human tissue and issues related to biological replication-should be clearly stated. Inclusion of any additional human data, if available, would substantially strengthen the manuscript

3- any potential issues raised by the reviewers related to statistical analysis and data presentation should be addressed

4-the reviewers encourage deeper interpretation of the main biological findings (i.e., the observed fibre type differences in myofibril size (Fig. 3D) and the potential vulnerability of type IIb fibres to ageing)

Thank you in advance for your kind consideration

Senior Editor:

Comments for Authors to ensure the paper complies with the Statistics Policy :
Please revisit The Journal's statistics policy to ensure full compliance. For example, in your revised version, please represent your data variability with SD rather than SEM. Thank you.

Comments to the Author:
Thank you for submitting your manuscript for consideration by The Journal of Physiology. As part of the peer review process, we recruited two Referees with expertise in this field of study. Both referees noted that there is potential for your manuscript to be influential in this field of study, however they also both noted some concerns that would need to be satisfactorily addressed for this impact to be realized. At this point we would like to invite you to respond point-by-point to each Referee's concerns, making corresponding revisions to your manuscript. Please also bring the revised paper into compliance with The Journal's statistics policy. We look forward to seeing your revised work and appreciate your interest in The Journal of Physiology.

REFEREE COMMENTS

Referee #1:

I have evaluated your manuscript, and present here some points for your consideration.

Major comments

As I understand the CellProfiler analysis workflow, the inter-myofibrillar area within a given muscle fibre is subtracted from

the cross-sectional area of that fibre, leaving the total myofibrillar area. This value is then divided by the mean myofibril area to estimate the number of myofibrils within that fibre. Thus, myofibril number appears to be calculated rather than directly counted. Please clarify whether this interpretation is correct. If so, I would appreciate further explanation of the rationale for estimating rather than directly counting myofibrils, as direct counting would appear feasible from the images shown. Have the authors tested whether directly counted and calculated myofibril numbers provide comparable results? This would seem important, given the central role of myofibril number in the manuscript.

Related to this, at the muscle-fibre level, both fibre size and fibre shape influence tissue architecture and how fibres can be packed within a given area (e.g. PMID: 26598531 and 38123165). In simple terms, fibre size matters, but so does geometry. Could similar principles apply at the myofibril level? For example, might myofibril shape, in addition to myofibril area, influence packing density or the interpretation of calculated myofibril number?

The validation of the high-resolution microscopy method is critical for the interpretation of the manuscript. At line 321, the authors refer to "indistinguishable results." Please be more specific here, as the accuracy of this approach relative to gold-standard electron microscopy is a key premise of the study. I appreciate the extensive methodological work presented previously by Jorgensen et al. However, as far as I can determine, the published direct comparison against electron microscopy was performed in mouse rather than human muscle tissue. Furthermore, the sample size in the direct comparison appears to reflect numbers of fibres rather than numbers of independent animals. As the biological unit of interest is the animal or human participant, this should be interpreted with some caution. I do not expect the authors to remedy limitations of a previously published validation study within the current manuscript, but the absence of direct validation in human tissue should be acknowledged, particularly as the present study reports differences in age-related patterns between humans and mice. Should the authors have additional validation data in human samples, their inclusion would substantially strengthen the manuscript.

The statistical analysis is generally clearly described and thoughtfully applied. However, I do not find the use of Fisher's LSD test ideal in this setting, as it does not adequately control for multiple comparisons. I do not necessarily suggest that the analyses be repeated, but borderline significant post hoc findings should be interpreted cautiously, and this limitation should be stated clearly. I also find the graphical presentation of main effects confusing. I appreciate that the authors report main effects, which is important and frequently omitted. However, the use of horizontal bars with associated P-values above individual groups may be interpreted as representing specific post hoc comparisons, even when the result reflects only a main effect in the absence of an interaction. This is particularly problematic in Fig. 4C-E, and especially Fig. 4E, where the figure may be read as showing direct differences between the two male groups and the two female groups. I suggest reporting the main effects in the same text box as the interaction term, rather than presenting them graphically using comparison bars.

The main effect of fibre type on myofibril size in Fig. 3D also deserves greater attention. As presented, type I fibres appear to contain larger myofibrils than type II fibres. In young individuals, type I and type II muscle-fibre cross-sectional areas appear largely similar, whereas the larger myofibril size in type I fibres seems to be accompanied by a numerically lower number of myofibrils. Did this finding surprise the authors? What might explain larger myofibrils in type I than type II fibres? I believe this is an interesting observation that warrants more discussion.

Similarly, the statement in lines 435-436 that highly glycolytic/type IIb fibres may exhibit a distinct vulnerability to ageing is intriguing and potentially important. However, in the absence of fibre type composition analyses in the mouse samples, could some of the observed age-related differences be explained by differences in type II fibre isoform composition? Type IIb fibres are substantially larger than both type IIx and type IIa fibres, and an age-related shift in fibre type composition could therefore potentially contribute to the observed differences in myofibril size in mice. Such fibre type shifts may be less pronounced in the human participants included in this study. I would encourage the authors to go further into this.

Finally, for the MRI analyses, the manuscript states that each of the four quadriceps muscles was quantified and summed to obtain total quadriceps muscle volume. While this is entirely acceptable, I am unclear as to why the vastus lateralis is not also presented separately, given that this is the muscle subsequently investigated at the microscopic and ultrastructural levels. Presenting vastus lateralis volume would provide a more direct link between the whole-muscle imaging data and the tissue-level analyses.

Minor comments

• Line 302 and throughout: the term "reduction" is not appropriate when describing cross-sectional differences, as it implies within-individual change over time. Please revise to terms such as "lower" or "smaller," depending on context.

• In accordance with journal policy, please present mean and SD, rather than SEM, in the figures.

• I recommend referring to fibres as type I and type II in the figures, rather than SERCA2 and SERCA1. For readers who are not familiar with SERCA-based fibre typing, particularly the inverse relation between SERCA nomenclature and conventional fibre type terminology, the current labelling is confusing.

• For both the muscle-fibre and myofibril analyses, please report the mean {plus minus} SD number of fibres and myofibrils analysed per sample.

• The Fig. 3 legend states "N = muscle/group." Please clarify whether this refers to individual participants/animals.

• Lines 289-290: please state how many "deviating samples" were excluded, and from which groups.

• Lines 79-80 state that skeletal muscle accounts for approximately 35% of total body mass, whereas the previous Jorgensen et al. 2024 paper refers to approximately 45%. I recognise that such estimates vary according to population and methodology, but I recommend aligning these statements.

• The Introduction is generally clear and well focused. However, line 123 states that the relationship between myofibre and myofibril adaptations is "largely unresolved," which suggests that some relevant data may already exist. If there are previous studies examining myofibre-myofibril interrelations during atrophy, please introduce this literature more explicitly and clarify the remaining knowledge gap addressed by the present study.

• Please clarify the time interval between visit 1 and visit 2. In particular, could the muscle biopsy have influenced the subsequent MRI assessment?

• Lines 172-173 refer to phosphate buffer (PB), whereas line 222 refers to phosphate-buffered saline (PBS) with Triton added. Please clarify whether these are different buffers or whether the terminology should be standardised.

• Line 234: please provide the manufacturer and catalogue/reference number for the No. 1 glass slides used.

Referee #2:

JP-RP-2026-291728 Myofibrillar Number

Introduction

The purpose of this paper was to measure both muscle fiber size, and myofibrillar number in skeletal muscles subjected to a altered trophic influences - in one case, the muscle atrophy is due to aging, and in another case, the atrophy is due to cast immobilization. The study includes both animal and human models, and clearly shows that the majority of the change in muscle fiber size is due to the change in myofibrillar number. This is an important finding. Overall, the experimental data are convincing, and the paper is framed in a proper historical context. The collaboration amongst the investigators is also impressive. In my opinion, this is a very nice contribution to the literature. My specific, minor comments are shown below.

Specific Comments

Line 32: space what exactly is "next generation" about the myofibrillar imaging pipeline? Or is it the MRI imaging?

Line 62: why were SERCA1 and SERCA2 used as the immunohistochemical markers?

Line 108: did the references provided rigorously show loss in fiber number with age?

Line 141: isn't proton MRI redundant? Shouldn't it just be MRI?

Line 151: having the subjects remain supine in for 20 minutes prior to scanning was a very good idea. Did this arise based on pilot data or is it simply suggesting that postural shifts and fluid volume would affect MRI values?

Line 171 what was the gauge of the biopsy needle?

Lines 244 to 254: making quantitative measurements from fluorescent images can be quite challenging because the fluorescent goes beyond the actual size of the structure. What evidence do you have that the myofibril size measured is true

and not artificially small based on the "size" of the fluorescent image?

Line 259:15 to 30 fiber seems like an extremely small number. Is there a reason why this value was chosen? Typically hundreds of fibers would be used to provide estimate of the true fiber size. Perhaps the 15 to 30 was to make sure that the fiber size of those 15 to 30 could also have all of the myofibrils measured?

Line 289: I'm confused about your definition of an outlier. "Samples that derived more than three times from the mean..." Three times what? It seems like it should be three standard deviations from the mean.

Line 313: I disagree with this point that you can say there was radial atrophy of the muscle fibers since you have an interaction term that's significant. It may be a detail, but really you have no main effect and therefore cannot make a general statement about radial atrophy.

Line 323: as above, you can't make the statement since you have a significant interaction term.

Line 344 it would be helpful here to say what the three factors are of the three-way nova. It does become clear later in the paragraph but perhaps this would be helpful earlier.

Nine 349: maybe the work on the TA could be put in a supplement?

By the way, figure 6 does not look like the cross-section of a soleus, but looks like the cross-section of a tibialis anterior muscle, complete with the "notch" for the EDL and the superficial curved surface. You might have made a mistake here

Line 284: what is the significance of the aggregates? Are they really relevant to the current study?

END OF COMMENTS

JP-RP-2026-291728 Myofibrillar Number

Introduction

The purpose of this paper was to measure both muscle fiber size, and myofibrillar number in skeletal muscles subjected to a altered trophic influences – in one case, the muscle atrophy is due to aging, and in another case, the atrophy is due to cast immobilization. The study includes both animal and human models, and clearly shows that the majority of the change in muscle fiber size is due to the change in myofibrillar number. This is an important finding. Overall, the experimental data are convincing, and the paper is framed in a proper historical context. The collaboration amongst the investigators is also impressive. In my opinion, this is a very nice contribution to the literature. My specific, minor comments are shown below.

Specific Comments

Line 32: space what exactly is “next generation” about the myofibrillar imaging pipeline? Or is it the MRI imaging?

Line 62: why were SERCA1 and SERCA2 used as the immunohistochemical markers?

Line 108: did the references provided rigorously show loss in fiber number with age?

Line 141: isn’t proton MRI redundant? Shouldn’t it just be MRI?

Line 151: having the subjects remain supine in for 20 minutes prior to scanning was a very good idea. Did this arise based on pilot data or is it simply suggesting that postural shifts and fluid volume would affect MRI values?

Line 171 what was the gauge of the biopsy needle?

Lines 244 to 254: making quantitative measurements from fluorescent images can be quite challenging because the fluorescent goes beyond the actual size of the structure. What evidence do you have that the myofibril size measured is true and not artificially small based on the “size” of the fluorescent image?

Line 259:15 to 30 fiber seems like an extremely small number. Is there a reason why this value was chosen? Typically hundreds of fibers would be used to provide estimate of the true fiber size. Perhaps the 15 to 30 was to make sure that the fiber size of those 15 to 30 could also have all of the myofibrils measured?

Line 289: I’m confused about your definition of an outlier. “Samples that derived more than three times from the mean…” Three times what? It seems like it should be three standard deviations from the mean.

Line 313: I disagree with this point that you can say there was radial atrophy of the muscle fibers since you have an interaction term that’s significant. It may be a detail, but

really you have no main effect and therefore cannot make a general statement about radial atrophy.

Line 323: as above, you can’t make the statement since you have a significant interaction term.

Line 344 it would be helpful here to say what the three factors are of the three-way nova. It does become clear later in the paragraph but perhaps this would be helpful earlier.

Nine 349: maybe the work on the TA could be put in a supplement?

By the way, figure 6 does not look like the cross-section of a soleus, but looks like the cross-section of a tibialis anterior muscle, complete with the “notch” for the EDL and the superficial curved surface. You might have made a mistake here

Line 284: what is the significance of the aggregates? Are they really relevant to the current study?

Responses to Editor and Referee Comments

EDITOR COMMENTS

The manuscript has now been reviewed by two reviewers expert in the topic. Both reviewers (an myself as Reviewing Editor) found the study to be well executed and a likely important contribution to the field. Specifically, the application of high-resolution microscopy to quantify myofibrillar number was viewed likely to have broad impact.

In preparing your revision, I suggest you to carefully address the following key points raised by the reviewers:

1- clarification and validation of the myofibril quantification approach - Reviewer 1 requests a clearer description of how myofibril number is derived from image analysis, specifically whether it is calculated rather than directly counted. If so, please justify this approach

2- the methodological validation of the high-resolution imaging technique should be more explicitly discussed. While prior validation against electron microscopy is acknowledged, limitations-especially the lack of direct validation in human tissue and issues related to biological replication-should be clearly stated. Inclusion of any additional human data, if available, would substantially strengthen the manuscript

3- any potential issues raised by the reviewers related to statistical analysis and data presentation should be addressed

4-the reviewers encourage deeper interpretation of the main biological findings (i.e., the observed fibre type differences in myofibril size (Fig. 3D) and the potential vulnerability of type IIb fibres to ageing).

We have addressed each of the points raised above in our response to the referees, and we have revised the manuscript accordingly. The referees' critiques were clear and constructive, and we believe we have resolved all outstanding concerns. It bears noting that an extensive amount of the revisions including generation of new figure etc.) were conducted by a postdoctoral fellow (Jared Hartung) in my lab that was not previously listed as an author. He is not included in the author list. Thank you for the opportunity to revise our manuscript.

.

SENIOR EDITOR COMMENTS

Comments for Authors to ensure the paper complies with the Statistics Policy: Please revisit The Journal's statistics policy to ensure full compliance. For example, in your revised version, please represent your data variability with SD rather than SEM. Thank you.

We have reviewed the policy and confirmed that all of requirements within it have been met in our revised manuscript.

Comments to the Author: Thank you for submitting your manuscript for consideration by The Journal of Physiology. As part of the peer review process, we recruited two Referees with expertise in this field of study. Both referees noted that there is potential for your manuscript to be influential in this field of study, however they also both noted some concerns that would need to be satisfactorily addressed for this impact to be realized. At this point we would like to invite you to respond point-by-point to each Referee's concerns, making corresponding revisions to your manuscript. Please also bring the revised paper into compliance with The Journal's statistics policy. We look forward to seeing your revised work and appreciate your interest in The Journal of Physiology.

Thank you for the opportunity to revise our manuscript. We hope that you and the referees will now find it to be acceptable for publication.

REFEREE #1 COMMENTS

As I understand the CellProfiler analysis workflow, the inter-myofibrillar area within a given muscle fibre is subtracted from the cross-sectional area of that fibre, leaving the total myofibrillar area. This value is then divided by the mean myofibril area to estimate the number of myofibrils within that fibre. Thus, myofibril number appears to be calculated rather than directly counted. Please clarify whether this interpretation is correct. If so, I would appreciate further explanation of the rationale for estimating rather than directly counting myofibrils, as direct counting would appear feasible from the images shown. Have the authors tested whether directly counted and calculated myofibril numbers provide comparable results? This would seem important, given the central role of myofibril number in the manuscript.

You are correct: the CellProfiler workflow first determines the intermyofibrillar area and subtracts this value from the total fiber CSA. We then divide the resulting myofibrillar area by the mean myofibrillar CSA to obtain the number of myofibrils.

We have not performed direct counting of myofibrils with our CellProfiler workflow in FIM-ID. The reason for this is that the sarcoplasmic reticulum (i.e., the outer border of the myofibrils) is most prominent around the Z-disc, and the SERCA1/2 signal, which marks the sarcoplasmic reticulum, is strongest when the Z-disc lies within the focal plane of the image. As elegantly shown in PMID: 32709902, sarcomeres are not perfectly planar but instead adopt a helicoid conformation, meaning Z-discs across the width of the fiber do not remain fully aligned in a single plane. This leads to an intermittent decrease in the SERCA1/2 signal as the Z-discs come in and out of the focal plane and can result in gaps in the signal for the sarcoplasmic reticulum. Because of this, the outer border of many myofibrils cannot be confidently resolved, and this point is true with both EM and FIM-ID based imaging, see adjacent examples.

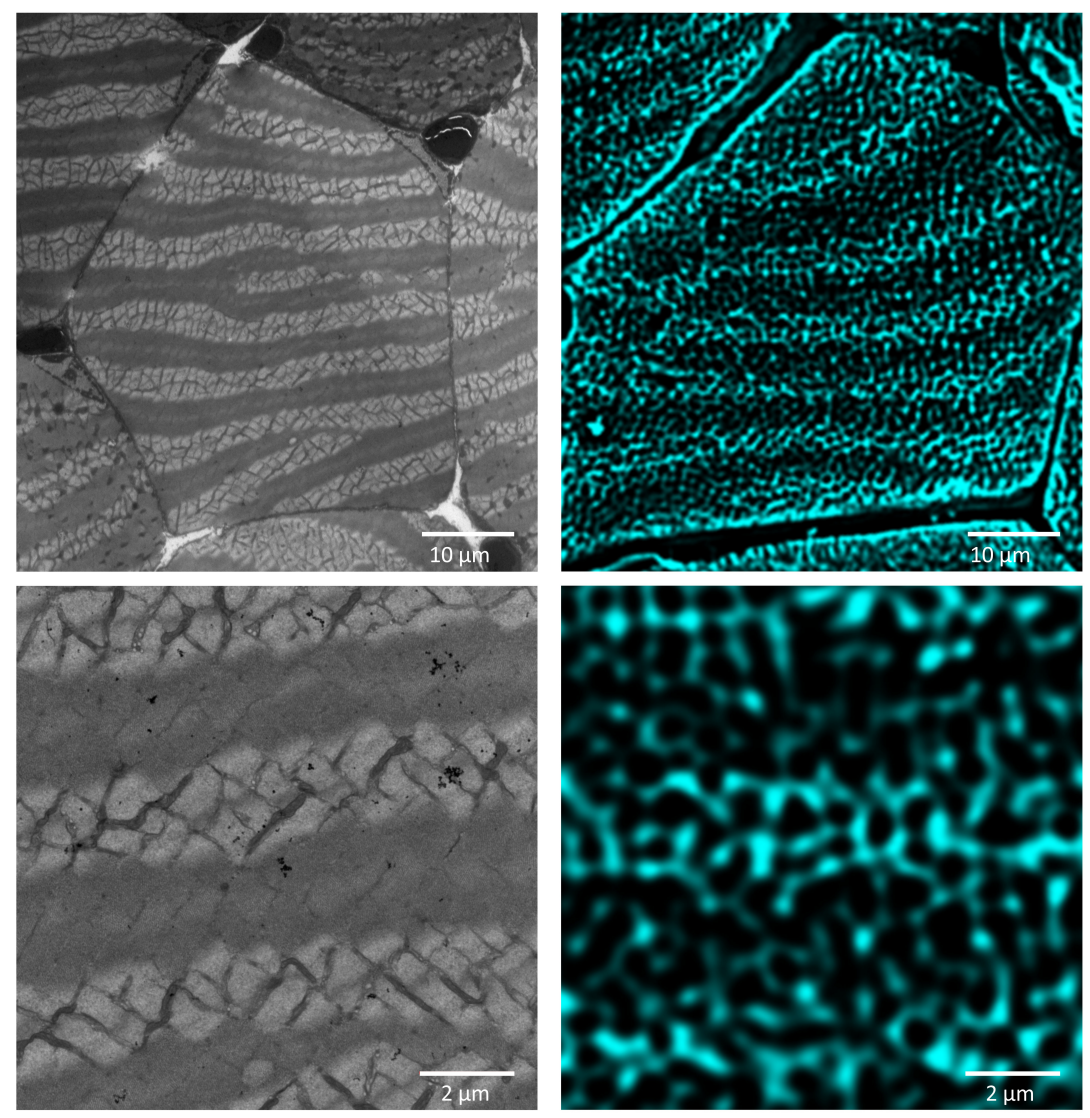

Representative images from EM (left) and FIM-ID for SERCA1 (right) of mouse skeletal muscle at lower and higher magnification.

Related to this, at the muscle-fibre level, both fibre size and fibre shape influence tissue architecture and how fibres can be packed within a given area (e.g. PMID: 26598531 and 38123165). In simple terms, fibre size matters, but so does geometry. Could similar principles apply at the myofibril level? For example, might myofibril shape, in addition to myofibril area, influence packing density or the interpretation of calculated myofibril number?

We agree that both myofibril shape and area can influence packing density. However, in our study, these variables should not impact the calculated myofibril number. The reason for this is that packing density is effectively the ratio of the myofibrillar area to the whole fiber cross-sectional area (CSA). Since we measure the myofibril area (whole fiber CSA - intermyofibrillar area), we are automatically correcting for any differences that could arise from packing density.

The validation of the high-resolution microscopy method is critical for the interpretation of the manuscript. At line 321, the authors refer to "indistinguishable results." Please be more specific here, as the accuracy of this approach relative to gold-standard electron microscopy is a key premise of the study. I appreciate the extensive methodological work presented previously by Jorgensen et al. However, as far as I can determine, the published direct comparison against electron microscopy was performed in mouse rather than human muscle tissue. Furthermore, the sample size in the direct comparison appears to reflect numbers of fibres rather than numbers of independent animals. As the biological unit of interest is the animal or human participant, this should be interpreted with some caution. I do not expect the authors to remedy limitations of a previously published validation study within the current manuscript, but the absence of direct validation in human tissue should be acknowledged, particularly as the present study reports differences in age-related patterns between humans and mice. Should the authors have additional validation data in human samples, their inclusion would substantially strengthen the manuscript.

As the reviewer mentioned, extensive validation work was conducted in Jorgensen et al. 2024 (PMID: 38466320). Indeed, the data in Figure 4 of that manuscript (particularly 4E and F) are what led us to state that the results with EM and FIM-ID are indistinguishable. Specifically, this data included samples from four mice per group and demonstrated that the EM vs. FIM-ID values for the myofibril CSA / fiber CSA ratio were within 4.2% of each other. Similarly, the values for the number of myofibrils per fiber CSA were within 3.6% of each other. Given these results, the description that the two methods produce indistinguishable results was, in our opinion, a fair description. Nevertheless, in recognition of the referees' comments, we have changed the description of the results from the two methods to 'highly concordant'. Furthermore, we appreciate the comment regarding the fact that the validation was only done in mouse skeletal muscles and have modified the text on line 327 to reflect this point.

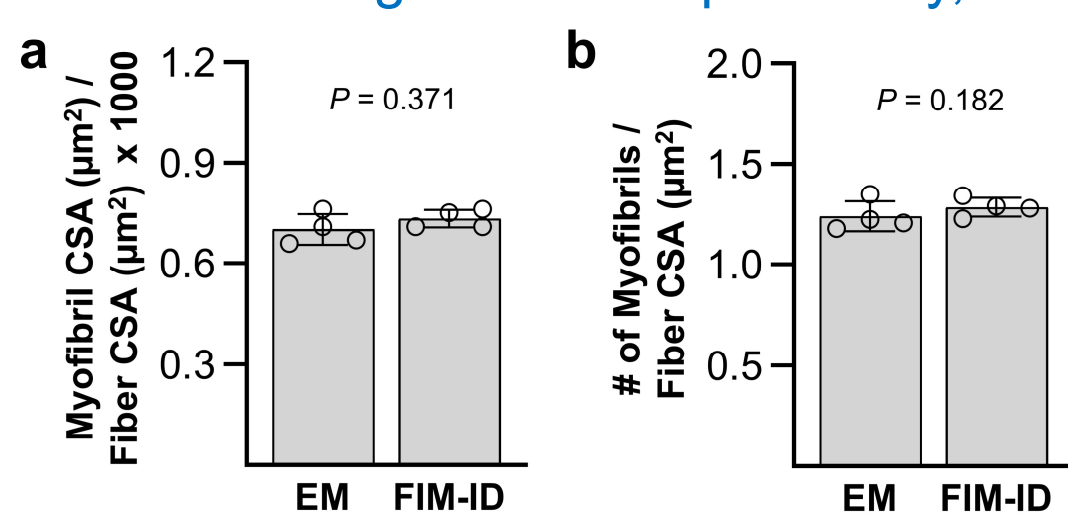

Data from Figure 4 of Jorgensen et. al (2024). For each mouse, one plantaris muscle was processed for imaging with EM, and the contralateral plantaris was processed for FIM-ID. The graphs illustrate (**a**) the relationships between myofibril CSA and fiber CSA, and (**b**) the number of myofibrils per fiber CSA. Data were derived from n = 4 muscles per group, and the mean value for each muscle was derived n = 36–40 fibers.

The statistical analysis is generally clearly described and thoughtfully applied. However, I do not find the use of Fisher's LSD test ideal in this setting, as it does not adequately control for multiple comparisons. I do not necessarily suggest that the analyses be repeated, but borderline significant post hoc findings should be interpreted cautiously, and this limitation should be stated clearly. I also find the graphical presentation of main effects confusing. I appreciate that the authors report main effects, which is important and frequently omitted. However, the use of horizontal bars with associated P-values above individual groups may be interpreted as representing specific post hoc comparisons, even when the result reflects only a main effect in the absence of an interaction. This is particularly problematic in Fig. 4C-E, and especially Fig. 4E, where the figure may be read as showing direct differences between the two male groups and the two female groups. I suggest reporting the main effects in the same text box as the interaction term, rather than presenting them graphically using comparison bars.

The referee is applauded for identifying some reporting errors that we made. Specifically, with respect to the use of Tukey's LSD, the original figures had instances in which *P* values were reported for comparisons that should not have been reported. Tukey's LSD was used for planned

comparison when the number of planned comparisons did not exceed k–1, where k is the number of groups (Figs. 3, 5, 6 and 8), and Sidak's was used when k-1 was exceeded (Fig. 7). The methods section has been updated to reflect this, and the graphs in the figures have been revised so that only *P* values for the planned comparisons are displayed. Also, as suggested, main effects are now reported as text instead of displayed as bars.

The main effect of fibre type on myofibril size in Fig. 3D also deserves greater attention. As presented, type I fibres appear to contain larger myofibrils than type II fibres. In young individuals, type I and type II muscle-fibre cross-sectional areas appear largely similar, whereas the larger myofibril size in type I fibres seems to be accompanied by a numerically lower number of myofibrils. Did this finding surprise the authors? What might explain larger myofibrils in type I than type II fibres? I believe this is an interesting observation that warrants more discussion.

As shown to the right, a similar finding was observed in the data we collected for Jorgenson et al. 2024 (PMID: 38466320). Hence, we were not surprised to see this again. In terms of an explanation, we can only speculate that the differences might be related to calcium diffusion. Specifically, a smaller myofibril CSA reduces the distance between the sarcoplasmic reticulum and the middle of the myofibril, allowing for quicker calcium diffusion. Additionally, as the myofibril CSA increases, the ratio of sarcoplasmic reticulum surface area to contractile area decreases linearly, further impairing calcium diffusion. Because Type II fibers are phenotypically differentiated from Type I fibers by their rapid twitch velocity, we hypothesize that the reduced myofibril CSA aids in maintaining rapid calcium diffusion, thereby facilitating the rapid twitch. We appreciate that the referee feels that this topic warrants more discussion, but anything we would say about this topic would be purely speculative. Since our study was not aimed at explaining these differences, we feel that such speculation would not be appropriate for the manuscript. Hopefully, the referee can understand our position on this.

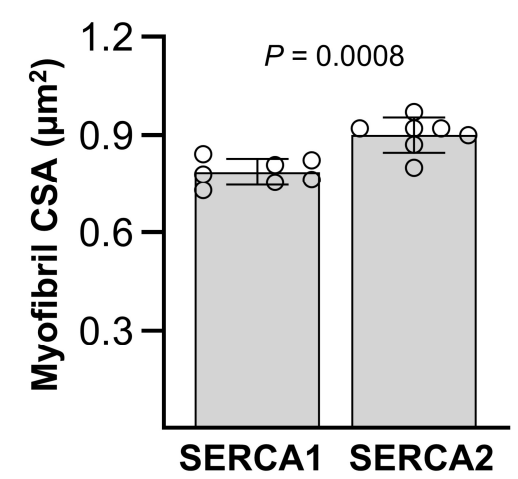

Myofibril cross-sectional area (CSA) comparison between SERCA1 and SERCA2 positive fibers obtained from human vastus lateralis biopsies in PMID: 38466320.

Similarly, the statement in lines 435-436 that highly glycolytic/type IIb fibres may exhibit a distinct vulnerability to ageing is intriguing and potentially important. However, in the absence of fibre type composition analyses in the mouse samples, could some of the observed age-related differences be explained by differences in type II fibre isoform composition? Type IIb fibres are substantially larger than both type IIx and type IIa fibres, and an age-related shift in fibre type composition could therefore potentially contribute to the observed differences in myofibril size in mice. Such fibre type shifts may be less pronounced in the human participants included in this study. I would encourage the authors to go further into this.

We are certainly open to the possibility that Type II fiber isoform shifts could have contributed to the age-related differences we observed. However, we would like to point out that similar results with aging were observed in both the soleus and FDL of mice. This bears mentioning because the soleus only contains a small number of Type 2X fibers and effectively no Type 2b fibers (PMID: 28892032 and 22530000). Hence, a potential Type 2b→2x→2a transition would not be expected to play a significant role in this muscle.

Finally, for the MRI analyses, the manuscript states that each of the four quadriceps muscles was quantified and summed to obtain total quadriceps muscle volume. While this is entirely acceptable, I am unclear as to why the vastus lateralis is not also presented separately, given that this is the muscle subsequently investigated at the microscopic and ultrastructural levels. Presenting vastus lateralis volume would provide a more direct link between the whole-muscle imaging data and the tissue-level analyses.

We thank the reviewer for this helpful suggestion. We agree that presenting vastus lateralis (VL) volume and CSA provides a more direct link between the MRI findings and the microscopic and ultrastructural analyses that we performed on biopsies from the VL. Accordingly, we have now included VL muscle volume and CSA data in the revised manuscript.

Minor comments

• Line 302 and throughout: the term "reduction" is not appropriate when describing cross-sectional differences, as it implies within-individual change over time. Please revise to terms such as "lower" or "smaller," depending on context.

This was viewed as a very fair point, and therefore, the term “reduction” has been eliminated from the description of the results.

• In accordance with journal policy, please present mean and SD, rather than SEM, in the figures.

Thank you for catching this oversight. All data are now presented as the mean and standard deviation.

• I recommend referring to fibres as type I and type II in the figures, rather than SERCA2 and SERCA1. For readers who are not familiar with SERCA-based fibre typing, particularly the inverse relation between SERCA nomenclature and conventional fibre type terminology, the current labelling is confusing.

We agree that the SERCA1/SERCA2 nomenclature will likely be less familiar to most readers than the conventional Type I/Type II terminology. However, it is important to note that the Type I and II fiber classification is conventionally assigned based on myosin heavy chain isoform expression, whereas fiber typing in our study was performed using SERCA immunodetection. We therefore retained the SERCA1/SERCA2 nomenclature to accurately reflect the methodology rather than adopt terminology based on a different molecular marker. To improve clarity, we have revised the manuscript to indicate that, in human skeletal muscle, SERCA2-positive fibers generally correspond to slow Type I fibers and SERCA1-positive fibers generally correspond to fast Type II fibers.

• For both the muscle-fibre and myofibril analyses, please report the mean {plus minus} SD number of fibres and myofibrils analysed per sample.

All relevant figure legends contain the range for the number of independent muscles per group, the number of analyzed fibers per muscle, and the number of myofibrils per fiber. In our opinion, reporting means +/- SD is not typically used when reporting information that is relevant to *n*.

• The Fig. 3 legend states "N = muscle/group." Please clarify whether this refers to individual participants/animals.

When applicable (including Figure 3), the wording (*n* = x-y muscles / group) now reads as (*n* = x-y independent muscles / group), to emphasize that all muscles were from different participants/animals. The only exception to this occurs in Figure 6, where the Soleus and FDL muscles were obtained from the same mice and were therefore now fully independent.

• Lines 289-290: please state how many "deviating samples" were excluded, and from which groups.

The simplest solution we could come up with to satisfy the request was to include the source data for all results presented in the study. In the source data excel file, we have included all the original values (including the outliers), and the values that were determined to be outliers have been highlighted in italics, along with an 'x' placed in front of them.

• Lines 79-80 state that skeletal muscle accounts for approximately 35% of total body mass, whereas the previous Jorgensen et al. 2024 paper refers to approximately 45%. I recognise that such estimates vary according to population and methodology, but I recommend aligning these statements.

The statements in the different studies are worded differently and were targeted with slightly different points of emphasis. The current statement is specific to the human body, and the supporting reference we selected indicates that 35% is the most appropriate number for this statement.

• The Introduction is generally clear and well focused. However, line 123 states that the relationship between myofibre and myofibril adaptations is "largely unresolved," which suggests that some relevant data may already exist. If there are previous studies examining myofibre-myofibril interrelations during atrophy, please introduce this literature more explicitly and clarify the remaining knowledge gap addressed by the present study.

To avoid any confusion, the wording "largely unresolved" has been changed to "unresolved".

• Please clarify the time interval between visit 1 and visit 2. In particular, could the muscle biopsy have influenced the subsequent MRI assessment?

The order of the MRI and muscle biopsy visits was determined by scanner availability and participant scheduling and therefore varied between participants. We have clarified the variability in the order of the study visits in the revised Methods. In participants who underwent the muscle biopsy before the MRI, the MRI assessment was performed at least 4 days after the biopsy. In those participants, the biopsy site was not visibly identifiable on the MRI images. Furthermore, the biopsy was obtained using a 6-mm Bergström needle, whereas muscle volume was quantified from contiguous 7-mm-thick MRI slices spanning the entire muscle. Therefore, any localized tissue disruption associated with the biopsy would have been confined to, at most, one or two image slices and would be expected to have a negligible influence on the overall muscle volume measurements.

• Lines 172-173 refer to phosphate buffer (PB), whereas line 222 refers to phosphate-buffered saline (PBS) with Triton added. Please clarify whether these are different buffers or whether the terminology should be standardised.

These are different buffers.

• Line 234: please provide the manufacturer and catalogue/reference number for the No. 1 glass slides used.

The details have been added.

REFEREE #2 COMMENTS

The purpose of this paper was to measure both muscle fiber size, and myofibrillar number in skeletal muscles subjected to a altered trophic influences - in one case, the muscle atrophy is due to aging, and in another case, the atrophy is due to cast immobilization. The study includes both animal and human models, and clearly shows that the majority of the change in muscle fiber size is due to the change in myofibrillar number. This is an important finding. Overall, the experimental data are convincing, and the paper is framed in a proper historical context. The collaboration amongst the investigators is also impressive. In my opinion, this is a very nice contribution to the literature. My specific, minor comments are shown below.

Thank you for the generous and supportive comments, they meant a lot to the authors.

Specific Comments

Line 32: space what exactly is "next generation" about the myofibrillar imaging pipeline? Or is it the MRI imaging?

The wording “Next generation” appears in the abstract and key points summary. It is a concise way of referring to our fluorescence imaging of myofibrils and image deconvolution (FIM-ID) methodology that allows us to obtain high resolution images of myofibrils with a standard wide-field fluorescence microscope.

Line 62: why were SERCA1 and SERCA2 used as the immunohistochemical markers?

Immunohistochemistry for SERCA1 and SERCA2 were performed because these are proteins that are highly enriched in the sarcoplasmic reticulum and can therefore be used to identify the periphery of the individual myofibrils.

Line 108: did the references provided rigorously show loss in fiber number with age?

In our view, the cited references do not provide rigorous support for a loss of fiber number with aging, and we regard this as a question that remains genuinely open. It was for precisely this reason that we chose our wording carefully: "At the macroscopic level, numerous studies have demonstrated that sarcopenia is marked by a decrease in whole muscle cross-sectional area (CSA), and that this effect **has been attributed** to coordinated changes at the microscopic level, including both the loss of muscle fibers (i.e., hypoplasia) and a reduction in the CSA of the muscle fibers (i.e., radial atrophy) (Lexell et al., 1988; Nilwik et al., 2013)." Our intent was to distinguish what has been claimed in the literature from what has been established. Lexell et al. (1988) has been cited more than 3400 times, and the large majority of those citations invoke the loss of fiber number reported in that study. We therefore maintain that it is accurate to state that the age-associated loss of muscle CSA has been attributed, at least in part, to a loss of fiber number. Whether this attribution rests on rigorous observations is debatable.

Line 141: isn't proton MRI redundant? Shouldn't it just be MRI?

While MRI can be performed using nuclei other than hydrogen, such as ^{13}Carbon or ^{31}Phosphorus, conventional clinical and research MRI refers to proton imaging unless otherwise specified. Therefore, we agree that the term "proton MRI" could be considered redundant in the present context and have revised the manuscript to remove the term “proton”.

Line 151: having the subjects remain supine in for 20 minutes prior to scanning was a very good idea. Did this arise based on pilot data or is it simply suggesting that postural shifts and fluid volume would affect MRI values?

The 20-minute supine rest period was not based on pilot data but was implemented to minimize the effects of postural fluid shifts on MRI-derived muscle size measurements. This approach was based on the findings of Berg *et al*. (1993) (PMID: 8213193), who demonstrated that thigh muscle cross-sectional area changes following the transition from standing to the supine position but remains stable after 20 minutes (see their Figure 3), indicating that standardizing body position before imaging minimizes variability associated with fluid redistribution.

Line 171 what was the gauge of the biopsy needle?

We have revised the Methods section to specify that muscle biopsies were obtained using a 6-mm Bergström biopsy needle with suction.

Lines 244 to 254: making quantitative measurements from fluorescent images can be quite challenging because the fluorescent goes beyond the actual size of the structure. What evidence do you have that the myofibril size measured is true and not artificially small based on the "size" of the fluorescent image?

We appreciate this question because we previously performed an extensive amount of work to validate FIM-ID against the gold standard method for visualizing myofibrils (i.e., electron microscopy). This work was published in 2024 (PMID: 38466320) and the bulk of the validation work can be found in Figure 4 of that study. In brief, one of the major points of validation for myofibril size came from the observation that there is no difference between the myofibril CSA to fiber CSA ratio observed with FIM-ID and electron microscopy. To assist the reviewer, this data is shown in the adjacent graph.

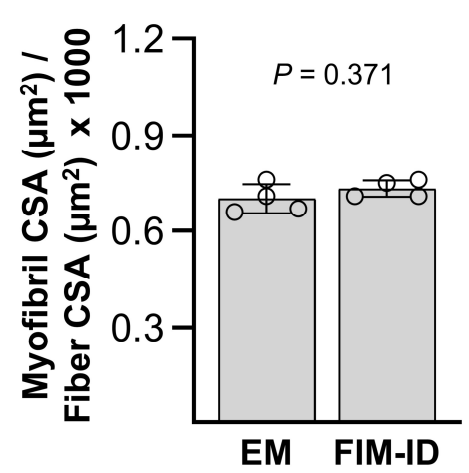

For each mouse, one plantaris muscle was processed for imaging with EM, and the contralateral plantaris was processed for FIM-ID, n=4 mice. The graph illustrates the relationships between myofibril cross-sectional area (CSA) and fiber CSA (n=144–160 fibers / group derived from 36–40 fibers /muscle).

Line 259:15 to 30 fiber seems like an extremely small number. Is there a reason why this value was chosen? Typically hundreds of fibers would be used to provide estimate of the true fiber size. Perhaps the 15 to 30 was to make sure that the fiber size of those 15 to 30 could also have all of the myofibrils measured?

We agree that 15-30 fibers would be too few to accurately estimate fiber CSA across an entire muscle cross-section. To clarify, the muscle fiber CSA values reported in Figures 3c, 4c, 6d, 8d, and A2b represent the mean CSA values of the randomly selected fibers that were processed for ultrastructural interrogation. In the revised manuscript, we have added a new figure (Figure A1) that presents the fiber CSA acquired with the Keyence microscope. Specifically, the Keyence allows us to capture 10x images of **all** fibers within a given sample, and these images were analyzed using our automated CellProfiler muscle fiber CSA pipeline (PMID: 34572107, see supplementary material for a detailed description). This pipeline measures the CSA of every qualifying fiber of a given fiber type and therefore enables us to obtain very strong estimates of the actual mean fiber CSAs for each sample. The 15 to 30 fibers referred to on line 259 are the ones that were randomly selected from the 63x FIM-ID images for subsequent myofibril-level analyses. As stated in the methods, the mean CSA of these fibers was compared to the mean obtained from the Keyence images, and when needed, additional randomly selected fibers were analyzed until the two means were within 10% (mouse) or 5% (human) of each other. In other words, our approach ensured that the fibers analyzed at the myofibril level had a mean CSA that was within 5-10% of the samples true mean. Importantly, the referee should note that there were

no differences in the conclusions derived from Keyence and FIM-ID based images (compare Figures 3c, 4c, 6d, 8d, and A2b, with the outcomes reported in Figure A1)

Line 289: I'm confused about your definition of an outlier. "Samples that derived more than three times from the mean..." Three times what? It seems like it should be three standard deviations from the mean.

We apologize, as the description of the method was poorly written. The sentence has been revised to read as "As previously described, values that were more than three SD's from the mean within a given group were excluded as outliers (Jorgenson et al., 2024)."

Line 313: I disagree with this point that you can say there was radial atrophy of the muscle fibers since you have an interaction term that's significant. It may be a detail, but really you have no main effect and therefore cannot make a general statement about radial atrophy.

We appreciate the point and agree that a general statement about radial atrophy would not be supported here. However, the sentence did not make a general statement, as the original text specified that the effect was fiber-type dependent and reported that CSA was unchanged in the SERCA2-positive fibers. We recognize, however, that the opening clause could have been read as a general claim before that qualification was reached. Hence, we have restructured the sentence so that the fiber-type dependence is stated first: "With respect to microscopic adaptations, the outcomes revealed that the effect of aging was dependent on fiber type. Specifically, aging led to a 23% decrease in the CSA of the SERCA1-positive fibers (i.e., radial atrophy), but it did not significantly alter the CSA of the SERCA2-positive fibers (Fig. 3c)".

Line 323: as above, you can't make the statement since you have a significant interaction term.

We respectfully disagree that a significant interaction precludes the statement we made, and we want to draw an important distinction between a main effect and a simple main effect. The significant interaction in Figure 3e ($P < 0.0001$) indicates that the effect of aging differed between the fiber types. It therefore precludes a general statement about all fibers (a point we accept and have addressed above). However, it does not preclude statements about the effect of aging within each fiber type. On the contrary, the significant interaction is what supports such a statement, as it is the formal evidence that the SERCA1-positive fibers responded differently from the SERCA2-positive fibers. Examining the effect of aging at each level of fiber type is the standard and recommended follow-up to a significant interaction (see Maxwell, Delaney & Kelley, Designing Experiments and Analyzing Data: A Model Comparison Perspective (3rd ed), 2018). Our sentence on line 323 reported exactly that "…. aging significantly reduced myofibril number in the SERCA1-positive fibers, but not in the SERCA2-positive fibers."

Line 344 it would be helpful here to say what the three factors are of the three-way nova. It does become clear later in the paragraph but perhaps this would be helpful earlier.

The sentence of concern has been rewritten to read as "To assess how immobilization and aging impacted muscle mass, the data were first subjected to a three-way ANOVA (i.e., age, immobilization, and muscle), and the outcomes revealed that there was a significant interaction between the effects of age and immobilization, and that the interaction was dependent on the muscle being examined ($P = 0.0003$)".

Line 349: maybe the work on the TA could be put in a supplement?

All of the data we obtained with the TA muscles was presented in the manuscript

By the way, figure 6 does not look like the cross-section of a soleus, but looks like the cross-section of a tibialis anterior muscle, complete with the "notch" for the EDL and the superficial curved surface. You might have made a mistake here

We thank the referee for their careful attention to this point and appreciate that the cross-section bears some resemblance to that of a TA. We can confirm, however, that the image is indeed from the soleus. First, no cross-sections of the TA were performed in this study. Second, there are two features that identify it is not a TA: its overall cross-sectional area and the number of fibers it contains, both of which are considerably smaller than would be expected for the TA.

Line 284: what is the significance of the aggregates? Are they really relevant to the current study?

In short, tubular aggregates are known, albeit poorly documented, formations of the sarcoplasmic reticulum that are seemingly only present in Type II fibers. Since our analytical methods relied on SERCA staining, the aggregates severely limit our ability to calculate myofibril number in fibers that had them. Hence acknowledging their existence and how we dealt with such fibers is critical to understanding the methods we employed in our study. Moreover, by documenting their presence in different conditions, our study adds to the limited body of knowledge that exists on them.

Dear Professor Hornberger,

Re: JP-RP-2026-291728R1 "**Macroscopic to Ultrastructural Analyses Identify the Loss of Myofibrils as the Primary Mediator of Aging- and Disuse-Induced Muscle Fiber Atrophy**" by Ramy K. A. Sayed, Anthony N Lange, Hector G Paez, Jamie E. Hibbert, Marius Meinhold, Corey G.K. Flynn, Jared Hartung, Matilde B.Z. Vergara Santibanez, Isabell Dobrzycki, David J. Wrucke, Carlos S. Zepeda, Jessica J. James, Christopher W. Sundberg, and Troy A. Hornberger

We are pleased to tell you that your paper has been accepted for publication in The Journal of Physiology.

The last Word (or similar) version of the manuscript provided will be used by the Production Editor to prepare your proof. When this is ready you will receive an email containing a link to Wiley's Online Proofing System. The proof should be thoroughly checked and corrected as promptly as possible.

Authors should note that it is too late at this point to offer corrections prior to proofreading. Major corrections at proof stage, such as changes to figures, will be referred to the Editors for approval before they can be incorporated. Only minor changes, such as to style and consistency, should be made at proof stage. Changes that need to be made after proof stage will usually require a formal correction notice.

All queries at proof stage should be sent to: TJP@wiley.com.

Following copyediting and typesetting the accepted version will be published online.

Yours sincerely,

Karyn Hamilton
Senior Editor
The Journal of Physiology

IMPORTANT POINTS TO NOTE FOLLOWING ACCEPTANCE OF YOUR PAPER:

• IMPORTANT NOTICE ABOUT OPEN ACCESS: To assist authors whose funding agencies mandate immediate public access to published research findings, The Journal of Physiology allows authors to pay an Open Access (OA) fee to have their papers made freely available immediately on publication.

The Corresponding Author will receive an email from Wiley with details on how to register or log in to Wiley Authors where you will be able to place an order.

• You can check if your funder or institution has a Wiley Open Access Account here:
https://authors.wiley.com/author-resources/Journal-Authors/open-access/author-compliance-tool.html

MEMBER DISCOUNT: Corresponding authors who are Members of the Physiological Society are eligible for a 20% discount on the cost of the Article Processing Charge (APC). To claim this discount, the Corresponding Author will need to log in to the Physiological Society Membership portal and retrieve the discount code (https://www.physoc.org/journals-and-media/journals/journal-discount-for-members/). This code should then be quoted in the billing section of Wiley Authors when selecting the open access option.

• TRANSPARENT PEER REVIEW POLICY: To improve the transparency of its peer review process, The Journal of Physiology publishes online as supporting information the peer review history of all articles accepted for publication. Readers will have access to decision letters, including Editors' comments and referee reports, for each version of the manuscript as well as any author responses to peer review comments. Referees can decide whether or not they wish to be named on the peer review history document.

• You can help your research get the attention it deserves! Check out Wiley's free Promotion Guide for best-practice recommendations for promoting your work at: www.wileyauthors.com/eeo/guide. You can learn more about Wiley Editing Services which offers professional video, design, and writing services to create shareable video abstracts, infographics, conference posters, lay summaries, and research news stories for your research at: www.wileyauthors.com/eeo/promotion.

• If you would like to receive our 'Research Roundup', a monthly newsletter highlighting the cutting-edge research published in The Physiological Society's family of journals (The Journal of Physiology, Experimental Physiology, Physiological Reports, The Journal of Nutritional Physiology and The Journal of Precision Medicine: Health and Disease), please click this link, fill in your name and email address and select 'Research Roundup':

https://www.physoc.org/journals-and-media/membernews

EDITOR COMMENTS

Reviewing Editor:

I thank the authors for their reply. I have no further comments.

Senior Editor:

Thank you for submitting your revised manuscript. Overall, the Referees were satisfied with the revisions and we are pleased to accept it for publication in The Journal of Physiology. Thank you for your interest in The Journal and Congratulations!

REFEREE COMMENTS

Referee #1:

No further comments.

Referee #2:

The authors have responded to my comments. In some cases, I think the responses were weak or simply argumentative. I don't think it materially affects the manuscript, but I would have liked more than one line denials of the point in some cases.

****
